# Steroid-sensitive nephrotic syndrome candidate gene *CLVS1* regulates podocyte oxidative stress and endocytosis

**DOI:** 10.1172/jci.insight.152102

**Published:** 2022-01-25

**Authors:** Brandon M. Lane, Megan Chryst-Stangl, Guanghong Wu, Mohamed Shalaby, Sherif El Desoky, Claire C. Middleton, Kinsie Huggins, Amika Sood, Alejandro Ochoa, Andrew F. Malone, Ricardo Vancini, Sara E. Miller, Gentzon Hall, So Young Kim, David N. Howell, Jameela A. Kari, Rasheed Gbadegesin

**Affiliations:** 1 Department of Pediatrics, Division of Nephrology, and Duke Molecular Physiology Institute, Duke University School of Medicine, Durham, North Carolina, USA.; 2 Pediatric Department, Pediatric Nephrology Center of Excellence, King Abdulaziz University, Jeddah, Saudi Arabia.; 3 Department of Biostatistics and Bioinformatics and Duke Center for Statistical Genetics and Genomics, Duke University, Durham, North Carolina, USA.; 4 Department of Medicine, Division of Nephrology, Washington University School of Medicine in St. Louis, St. Louis, Missouri, USA.; 5 Department of Pathology;; 6 Department of Medicine, Division of Nephrology; and; 7 Department of Molecular Genetics and Microbiology, Duke University School of Medicine, Durham, North Carolina, USA.

**Keywords:** Nephrology, Chronic kidney disease, Genetic diseases, Monogenic diseases

## Abstract

We performed next-generation sequencing in patients with familial steroid-sensitive nephrotic syndrome (SSNS) and identified a homozygous segregating variant (p.H310Y) in the gene encoding clavesin-1 (*CLVS1*) in a consanguineous family with 3 affected individuals. Knockdown of the clavesin gene in zebrafish (clvs2) produced edema phenotypes due to disruption of podocyte structure and loss of glomerular filtration barrier integrity that could be rescued by WT *CLVS1* but not the p.H310Y variant. Analysis of cultured human podocytes with CRISPR/Cas9-mediated *CLVS1* knockout or homozygous H310Y knockin revealed deficits in clathrin-mediated endocytosis and increased susceptibility to apoptosis that could be rescued with corticosteroid treatment, mimicking the steroid responsiveness observed in patients with SSNS. The p.H310Y variant also disrupted binding of clavesin-1 to **α**-tocopherol transfer protein, resulting in increased reactive oxygen species (ROS) accumulation in *CLVS1*-deficient podocytes. Treatment of *CLVS1*-knockout or homozygous H310Y-knockin podocytes with pharmacological ROS inhibitors restored viability to control levels. Taken together, these data identify *CLVS1* as a candidate gene for SSNS, provide insight into therapeutic effects of corticosteroids on podocyte cellular dynamics, and add to the growing evidence of the importance of endocytosis and oxidative stress regulation to podocyte function.

## Graphical abstract

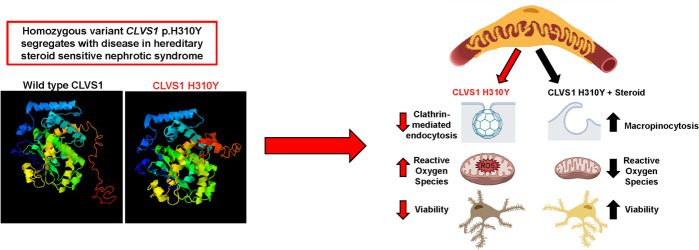

## Introduction

Childhood nephrotic syndrome (NS) is a common pediatric kidney disease estimated to affect 16/100,000 children worldwide (1). In children, 80% of all cases of NS are steroid responsive and are referred to as steroid-sensitive nephrotic syndrome (SSNS). The mechanisms by which corticosteroids cause remission in NS and their effects on podocyte function and viability are not completely understood.

NS due to single-gene defects is found in 10%–30% of patients depending on the population being studied (2–4). Most cases of genetic NS are steroid resistant and are often due to defects in glomerular visceral epithelial cell (i.e., podocyte) structural proteins (5, 6). Podocytes are an essential cellular component of the kidney glomerular filtration barrier (GFB). SSNS, on the other hand, is largely considered an immune-mediated disease; however, pathogenic variants in some genes involved in podocyte cytoskeletal dynamics have been reported in patients with SSNS (6–10). Moreover, while corticosteroids can act as immunosuppressants, they can also have protective effects on podocytes through stabilization of the actin cytoskeleton (11, 12).

In this study, we identify a homozygous variant (c.928C>T, p.H310Y, ENST00000519846.1, rs139500383) in the gene encoding clavesin-1 (*CLVS1*, NC_000008.11) as a potential cause of SSNS in a consanguineous family with 3 affected individuals. CLVS1 is expressed in multiple human tissues, including the kidney and brain; however, studies to date have been limited to characterizations of its function in neurons (13, 14). Clavesin-1 is required for proper endosome formation and is suspected of having a role in clathrin-mediated endocytosis (CME) based on its ability to bind clathrin and phosphatidylinositol 3,5-bisphosphate (13). Podocyte endocytosis is critical for the regulation of nephrin cycling and maintenance of the slit diaphragm, with several endocytosis-related genes having recently been established as contributors to podocyte dysfunction in NS (15–20).

Our analysis reveals that the zebrafish clavesin gene is required for the maintenance of GFB integrity in vivo. We validated the requirement for *CLVS1* in human podocyte homeostasis in vitro by demonstrating reduced viability and endocytosis in podocytes that are deficient in *CLVS1*; furthermore, we showed that this phenotype could be rescued with corticosteroid treatment. The p.H310Y variant was shown to be deleterious to clavesin-1 function in vitro and in vivo and affected binding to the ligand, α-tocopherol transfer protein. This reduced affinity for a transporter of the antioxidant α-tocopherol caused an increased accumulation of reactive oxygen species (ROS) in *CLVS1*-knockout and homozygous H310Y-knockin podocytes. Moreover, pharmacological inhibition of ROS accumulation was sufficient to rescue the aberrant viability phenotype in podocytes with reduced functional clavesin-1, revealing new possible therapeutic strategies for patients with NS due to *CLVS1* defects and a possible adjunct therapy for children with the more common idiopathic NS.

## Results

### A rare variant in CLVS1 is a likely cause of hereditary SSNS.

**Figure 1:**
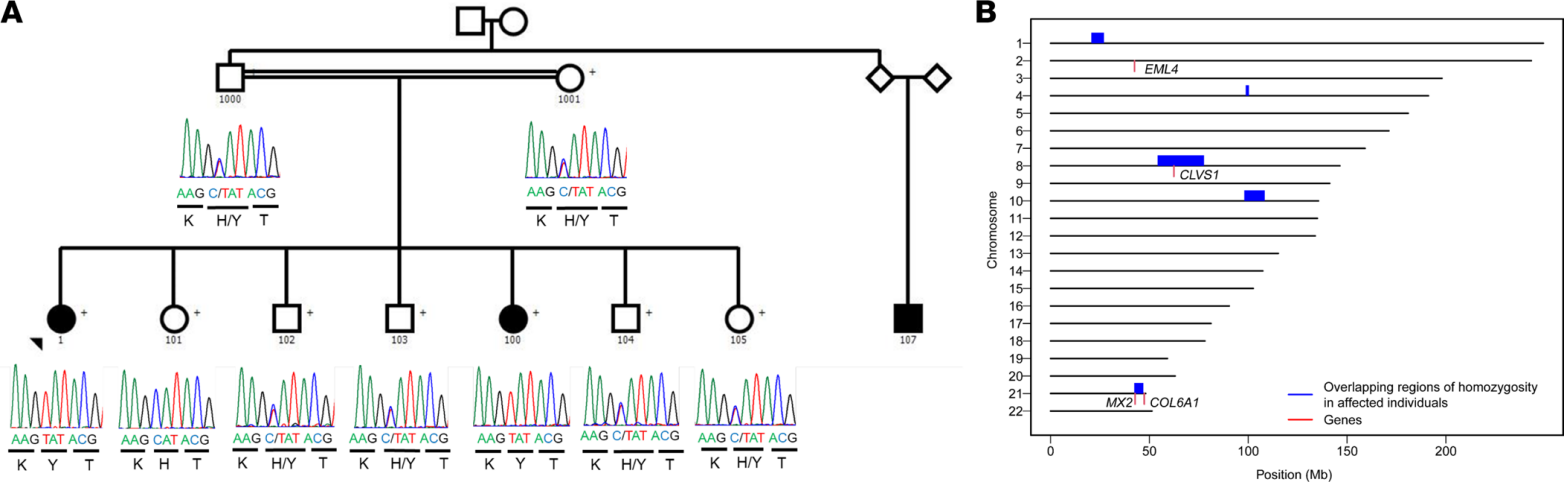
*CLVS1* p.H310Y is a potential cause of familial childhood SSNS. (**A**) Pedigree of family with SSNS showing segregation of the homozygous *CLVS1* p.H310Y variant (c.928C>T, GRCH37/hg19) with disease. Filled circles and square represent affected individuals; unfilled circles, squares, and diamonds represent unaffected individuals. The parents of the family were estimated from their sequencing data to be second degree relatives (kinship coefficient of 0.106). (**B**) Regions of homozygosity (ROH) potentially implicated in SSNS. The 5 ROH (blue boxes, spanning each of the ROH on its respective chromosome) are those shared by both children with SSNS (ID 1 and 100) and are not in ROH in any of the unaffected relatives who were sequenced (ID 1000, 1001, 101). Gene locations are marked with red vertical lines. Candidate genes *CLVS1* and MX2 are located in these ROH.

Next-generation sequencing (whole-genome or whole-exome sequencing) was carried out in 42 sibling pairs with SSNS in our efforts to identify single-gene causes of SSNS. We subjected the sequencing data from each family to an established filtering algorithm for identification of single-gene causes of NS (21). We identified a rare missense variant (c.928C>T, p.H310Y, NC_000008.11, rs139500383) in *CLVS1* as the only segregating variant candidate in family DUK40585 (Figure 1A, Supplemental Figure 1, and Supplemental Table 1; supplemental material available online with this article; https://doi.org/10.1172/jci.insight.152102DS1). We found homozygous variants in 3 other genes (*COL6A1*, *MX2*, *EML4*) that were present in affected individuals and unaffected siblings but heterozygous in both parents (Supplemental Table 1). Family DUK40585 consists of unaffected consanguineous parents with 3 affected individuals comprising 2 sisters and a paternal cousin (Figure 1A). All 3 affected individuals responded to corticosteroid treatment, although all 3 have a frequently relapsing and steroid-dependent course that was responsive to cyclophosphamide and rituximab (Table 1). Follow-up data at 8 and 2 years for individuals 1 and 100 showed that they were still in remission with normal kidney function (Table 1).

Homozygosity mapping of the affected family revealed that 5 significant regions of homozygosity (ROH) were present in both SSNS cases and absent in unaffected relatives (Figure 1B and Supplemental Table 2). The largest ROH, located on chromosome 8, contains the *CLVS1* gene. None of the known single-gene causes of autosomal-recessive NS are located in these ROH (Supplemental Table 2).

The *CLVS1* gene exhibits a high degree of genetic constraint in humans with significantly fewer loss-of-function mutations reported in the general population than predicted, indicating that the product of this gene likely has important biological functions (probability of loss of function intolerance = 0.854) (22, 23). The histidine residue at amino acid position 310 of clavesin-1 is highly conserved in evolution (Table 2), and the tyrosine substitution found in the p.H310Y variant was predicted to be deleterious to clavesin-1 function by multiple in silico analyses (Supplemental Table 1). This variant is not present in a homozygous state in 281,974 chromosomes in the Genome Aggregation Database (gnomAD) (22). Sequencing of *CLVS1* in 604 additional patients with familial and sporadic NS of unclear etiology did not reveal other potentially pathogenic variants, suggesting that mutations in *CLVS1* are likely to be a very rare cause of familial SSNS.

### Clavesin-1 is expressed in the podocyte.

The expression and function of clavesin-1 in most tissues, including the kidney, has yet to be fully defined. We used immunofluorescence staining and immunoblotting to reveal the expression and colocalization of *CLVS1* with *WT1* proteins in podocytes from extracted mouse glomeruli (Supplemental Figure 2, A and B). Although human podocyte *CLVS1* expression has already been reported in single-cell sequencing data collected from kidney tissue samples, we used immunoblotting to confirm the expression of *CLVS1* in conditionally immortalized human podocyte cell lines (Supplemental Figure 2, C–F) (24, 25). Despite relatively high *CLVS1* expression in neuronal cells compared with podocytes, neurological abnormalities have not been reported in the family carrying the p.H310Y variant, suggesting that clavesin-1 may have podocyte-specific functions that are critical for maintenance of the GFB.

### The zebrafish clavesin gene is required for maintenance of the GFB.

**Figure 2:**
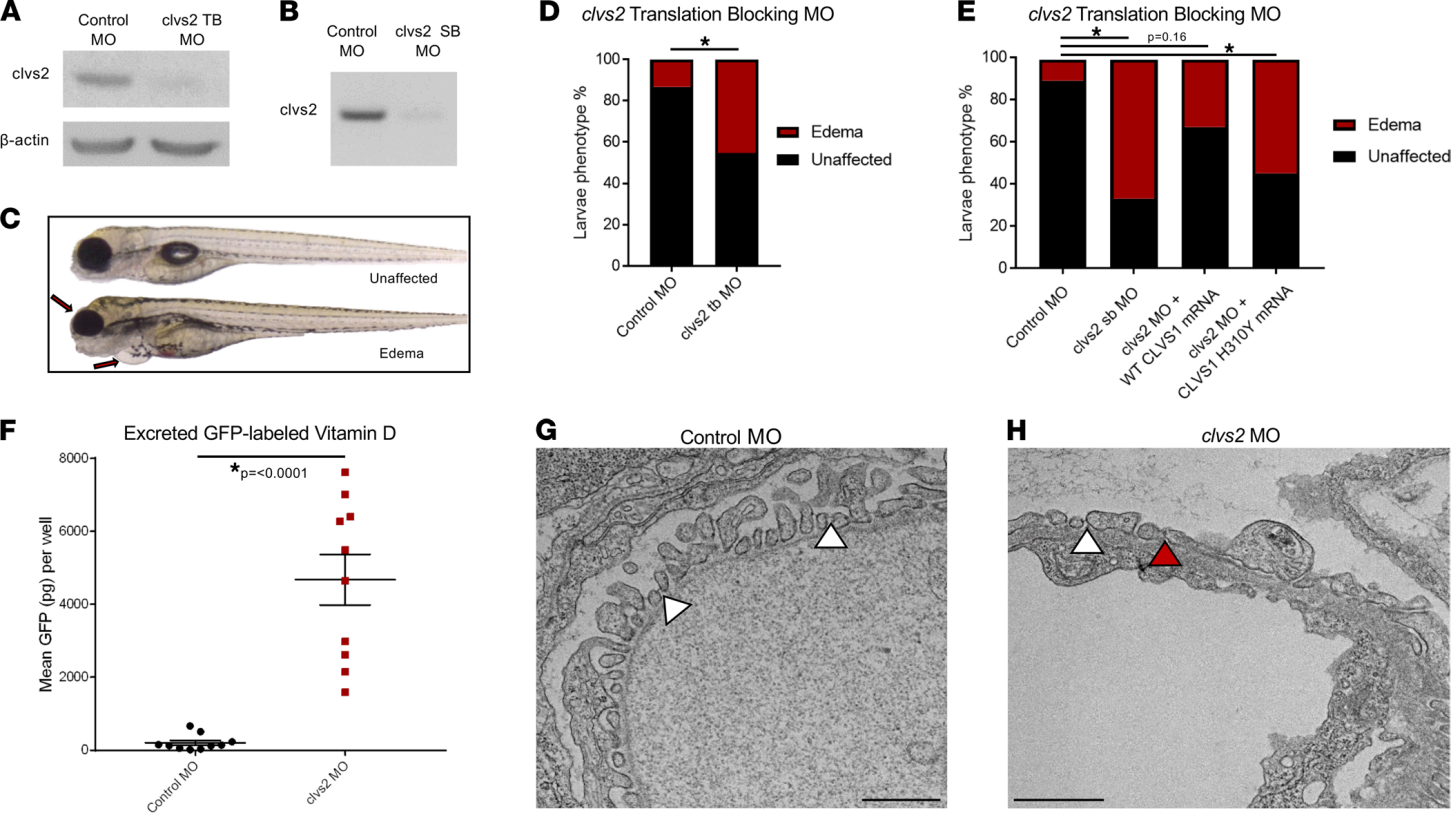
Knockdown of the clavesin gene in zebrafish (*clvs2*) results in edema phenotypes. (**A** and **B**) Translation blocking and splice block morpholinos were used to knock down *clvs2* expression in zebrafish. Morpholino efficacy was confirmed through Western blot (**A**) and reverse transcription PCR (RT-PCR) (**B**), showing that both morpholinos were able to knock down *clvs2* expression. (**C**) Larval phenotypes were evaluated at 4 days postfertilization (dpf) as having edema (arrows for periorbital and pericardial) or as unaffected (no edema). (**D** and **E**) Analysis revealed significantly increased edema phenotypes with *clvs2* knockdown compared with controls, and this edema could be rescued by coinjection of wild-type human *CLVS1* mRNA but not the p.H310Y variant (**E**) (*P < 0.05, *n* > 60 for all groups, 1-way ANOVA). (**F**) Quantification of excreted GFP-labeled vitamin D in the Tg(lfabp:VDBP-GFP) reporter line revealed a loss of GFP in clvs2 morpholino-injected fish compared with controls (*n* = 10 for each group, *P* < 0.0001, 2-tailed *t* test), demonstrating that GFB integrity was affected by clvs2 knockdown. (**G** and **H**) Transmission electron microscopy images show healthy podocyte foot processes with intact slit diaphragms (white arrowheads) around a capillary loop in 5 dpf control morphant larvae and podocyte effacement (red arrowhead) in *clvs2* morphants (scale bars = 800 nm and 1 μm, respectively), confirming that edema phenotypes are due to reduced GFB integrity.

As the p.H310Y variant is predicted to be damaging to clavesin-1 function, we examined the effects of reduced clavesin protein activity on glomerular function in vivo using morpholino-mediated gene knockdown. The single zebrafish clavesin gene, *clvs2*, is the ortholog of human *CLVS2*, the paralog of *CLVS1*. While the H310 residue is not conserved, *clvs2* contains the major functional domains of both *CLVS1* and *CLVS2*, which allowed us to evaluate the requirement for clavesin protein function in the maintenance of zebrafish GFB integrity. Knockdown of *clvs2* in zebrafish larvae using both translation blocking and splice blocking morpholinos was sufficient to induce edema phenotypes when compared with control morpholino-injected fish at 4 days postfertilization (Figure 2, A–E). We confirmed that the edema phenotypes in *clvs2* morpholino (MO) fish resulted from a disruption of the GFB by quantifying the excretion of fluorescently labeled vitamin D binding protein (a surrogate marker of albuminuria-sized protein leak) in the established Tg(lfabp:vdbp-GFP) reporter fish line (Figure 2F) (26). Podocyte foot process effacement was observed in *clvs2* MO larvae and not in control morphants (Figure 2, G and H). The edema phenotype in *clvs2* MO larvae could be rescued by the addition of exogenous zebrafish *clvs2* mRNA as well as wild-type human *CLVS1* mRNA but not by human *CLVS1* mRNA encoding the p.H310Y variant (Figure 2E). Taken together, these data suggest that there is an evolutionary requirement for clavesin proteins in the maintenance of GFB integrity and that the p.H310Y variant disrupts clavesin-1 function.

### Loss of CLVS1 decreases human podocyte viability that can be rescued by corticosteroid treatment.

**Figure 3:**
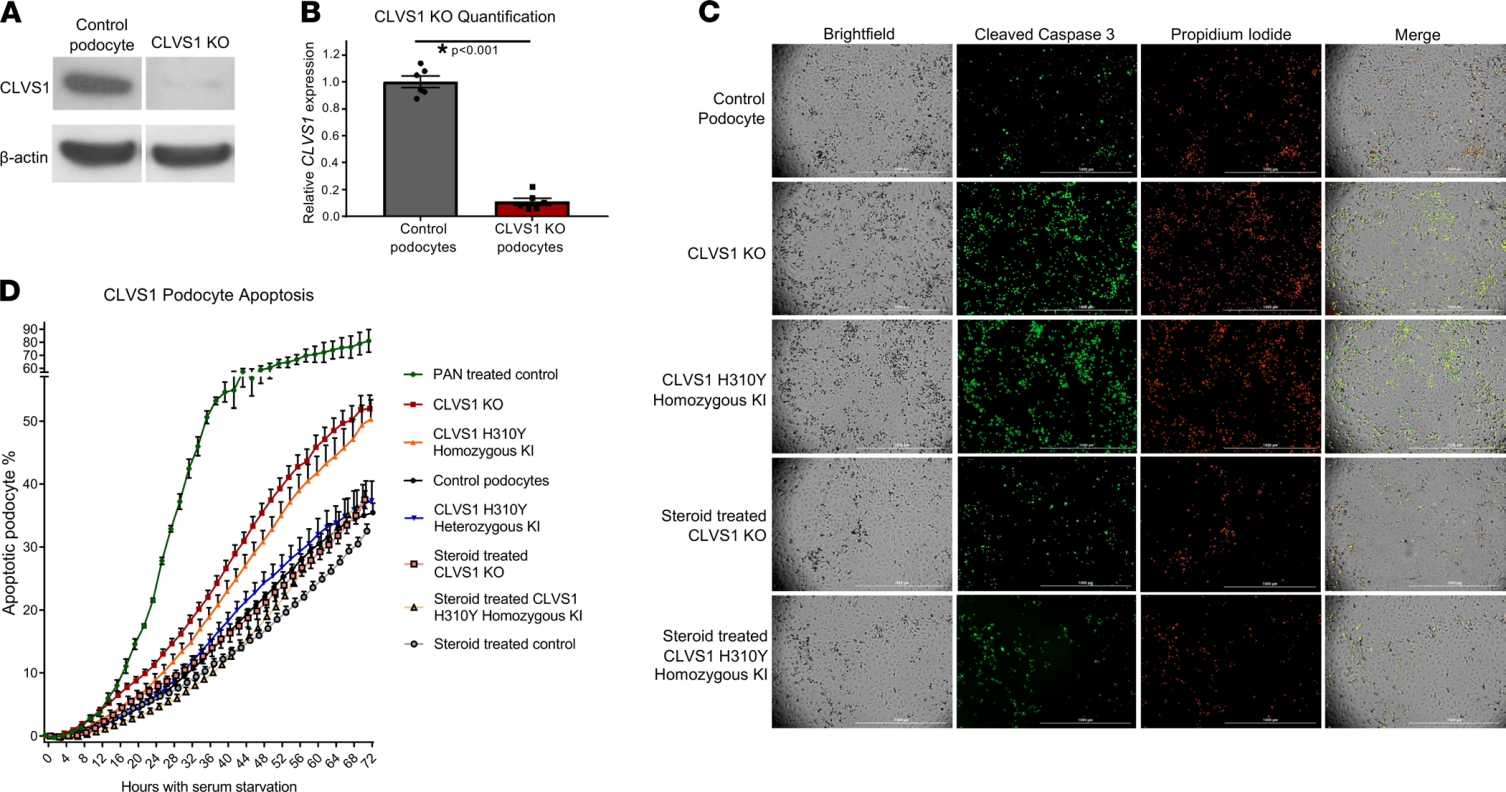
Loss of *CLVS1* expression increases podocyte susceptibility to apoptosis that can be restored by corticosteroid treatment. (**A** and **B**) Western blot showing significantly reduced expression of *CLVS1* in CRISPR/Cas9-mediated knockout (KO) podocyte cell lines compared with control cell lines (*n* = 4, *P* < 0.001, 2-tailed *t* test). (**C**) Still images depicting cleaved caspase-3 activity (green) as reporter for early apoptosis and propidium iodide staining (red) for late apoptosis and necrosis in human podocytes 72 hours after serum starvation (scale bars = 1 mm). (**D**) Quantification of these images over time revealed an increase in podocyte susceptibility to serum starvation–induced apoptosis in *CLVS1*-KO and homozygous H310Y-knockin (KI) podocytes compared with controls (*P* < 0.05 for all time points after 16 hours for KO and after 34 hours for KI, *n* > 25 for each group, 2-way ANOVA). Heterozygous H310Y-KI podocytes displayed similar apoptotic phenotypes to controls. The elevated apoptosis in *CLVS1*-KO and homozygous H310Y-KI podocytes was rescued by treatment with 1 μM dexamethasone.

To examine the requirement of functional clavesin-1 activity for the maintenance of human podocyte homeostasis, we created conditionally immortalized human podocyte cell lines with CRISPR/Cas9-mediated *CLVS1* knockout (KO). Additionally, to examine the specific effects of the H310Y variant, we used CRISPR/Cas9 gene editing to create heterozygous and homozygous *CLVS1* H310Y knockin (KI) podocyte lines. Automated live-cell imaging of cleaved caspase-3 enzymatic activity using a fluorescently labeled substrate of cleaved caspase-3 allowed for quantification of podocyte apoptosis over time (Supplemental Videos 1–3). Propidium iodide (PI) staining was used as a marker of late apoptosis and necrosis. When exposed to serum starvation over 72 hours, *CLVS1*-KO and homozygous H310Y-KI podocytes displayed significantly increased apoptosis and total cell death compared with untransfected podocyte controls (Figure 3 and Supplemental Figure 3). Heterozygous H310Y-KI podocytes displayed similar levels of apoptosis compared to controls, providing additional evidence for the role of *CLVS1* in the development of autosomal-recessive disease.

As the affected family with the *CLVS1* p.H310Y variant responded to corticosteroid treatment, we examined the effects of dexamethasone on *CLVS1* podocytes. We treated podocyte cell lines with 1 μM dexamethasone during 72 hours of serum starvation. The addition of dexamethasone eliminated the increased apoptosis in *CLVS1*-KO and homozygous KI podocytes compared with controls, mirroring the phenotype observed in the affected family (Figure 3 and Supplemental Figure 3). These same corticosteroid-responsive apoptosis phenotypes were confirmed in podocyte cell lines with stable lentiviral shRNA-mediated *CLVS1* knockdown (KD) as well as HEK293 cells overexpressing the H310Y variant compared with their respective controls (Supplemental Figures 4 and 5).

### CLVS1 is required for CME in human podocytes.

**Figure 4:**
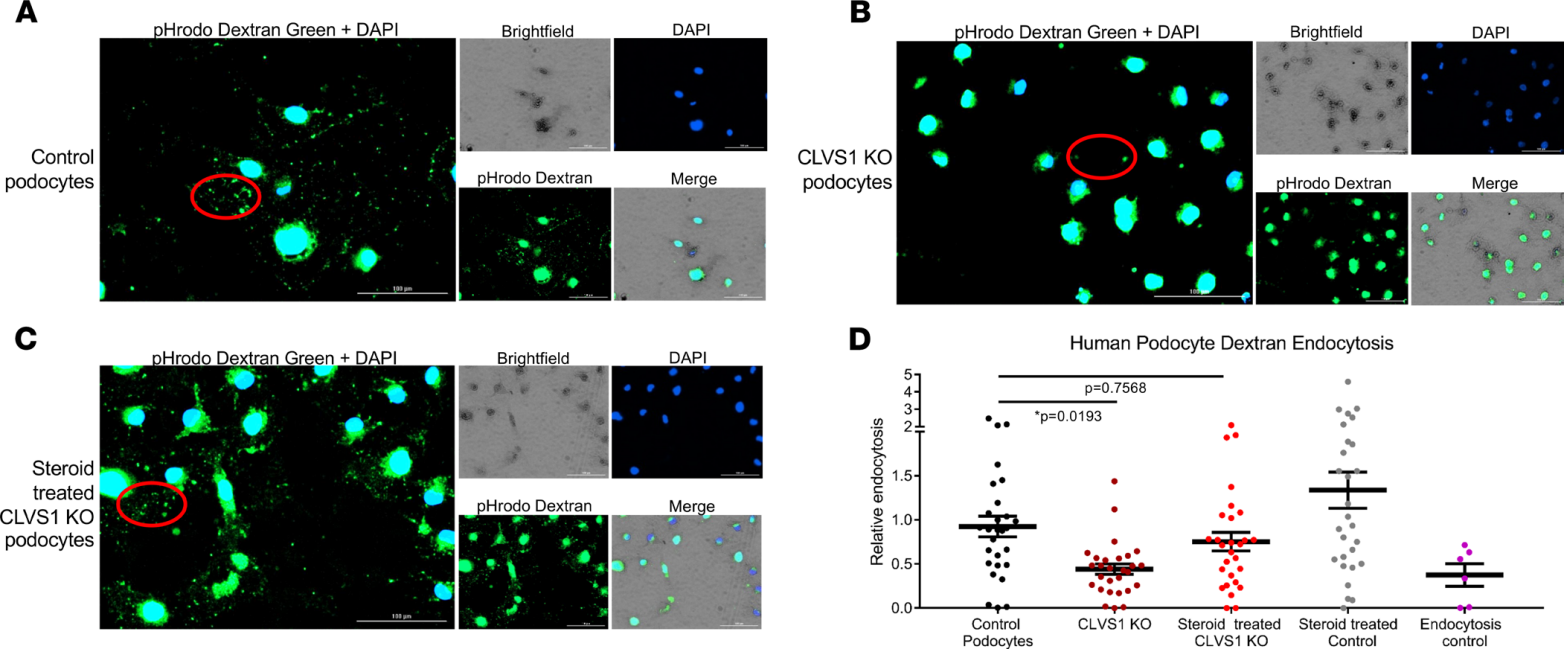
Loss of *CLVS1* causes decreased podocyte endocytosis. (**A**–**C**) Images showing the decreased endocytosis of fluorescently labeled dextran (pHrodo Dextran) in conditionally immortalized podocytes with CRISPR/Cas9-mediated KO of *CLVS1* that can be restored with treatment with 1 μM dexamethasone (representative molecules circled in red). (**D**) Quantification of these images revealed a significant (*P* = 0.0193) loss of dextran endocytosis in KO podocytes compared with controls that was eliminated when these podocytes were treated with dexamethasone (*P* = 0.7568, *n* > 20 for each experimental group, 1-way ANOVA). A selective inhibitor of dynamin I and dynamin II, Dynasore, which reduces clathrin-mediated endocytosis, was used as an endocytosis control. Errors bars depict SEM.

To understand the contributions of *CLVS1* deficits to reduced podocyte viability, we examined the impact on cellular endocytosis. Clavesin-1 has been shown to be required for proper endosome formation, and its ability to bind both clathrin and phosphatidylinositol 3,5-bisphosphate suggests a role in CME (13). We first examined podocyte endocytosis through the use of a 10,000 MW dextran compound (pHrodo) that is pH sensitive and fluoresces green when internalized within the cell. Internalized dextran molecules were quantified through automated live-cell imaging to compare the relative endocytosis between podocyte cell lines. *CLVS1*-KO podocytes displayed decreased endocytosis of dextran when compared with their respective controls, demonstrating the importance of *CLVS1* function to endocytic processing in podocytes (Figure 4 and Supplemental Figure 3). Pretreatment of *CLVS1*-KO podocytes with dexamethasone restored the endocytosis of dextran to untreated podocyte control levels. These findings were again confirmed in *CLVS1*-KD podocytes and HEK293 cells overexpressing the H310Y variant compared with controls (Supplemental Figures 4 and 5).

**Figure 5:**
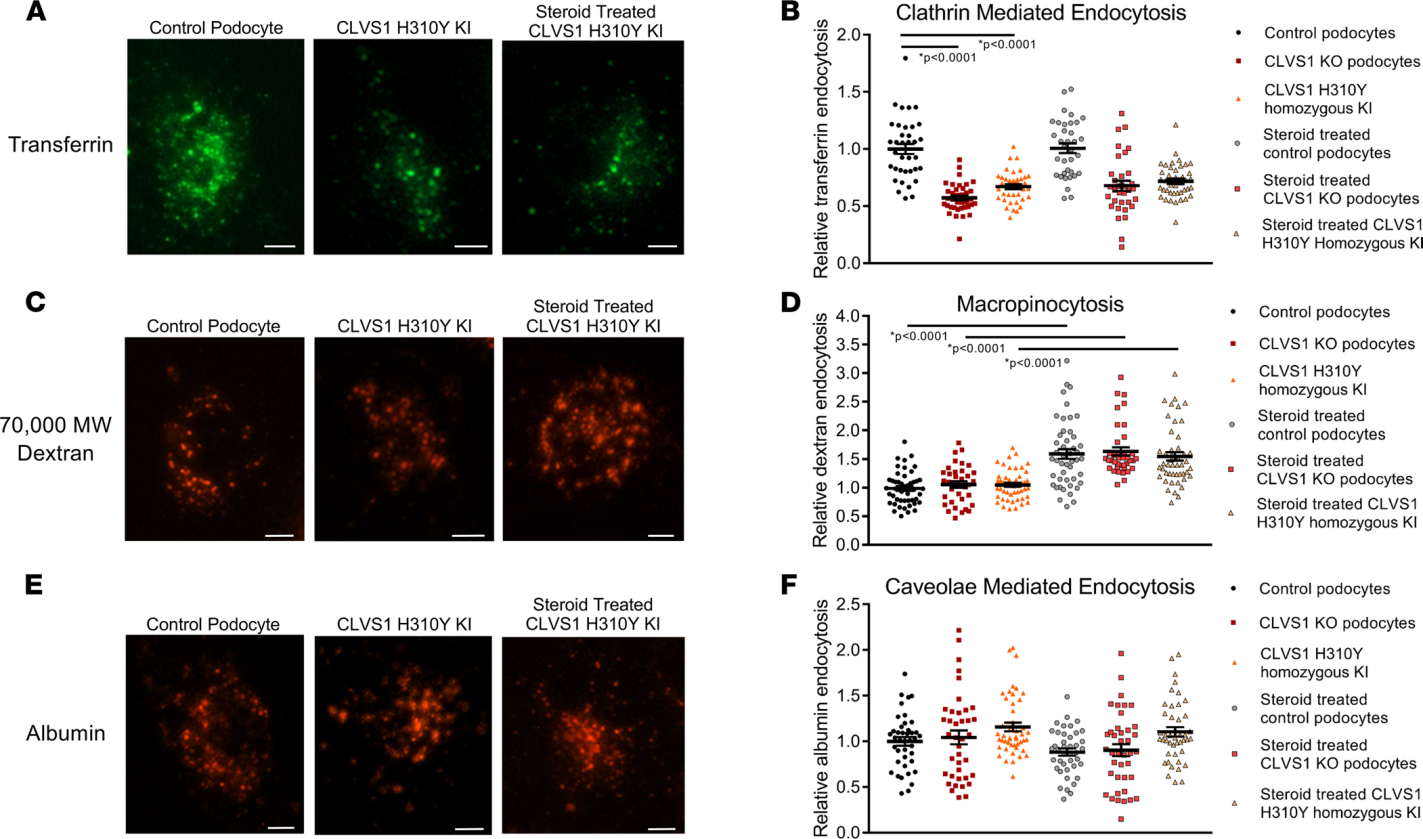
*CLVS1* is required for clathrin-mediated endocytosis in human podocytes. To examine specific modes of endocytosis in *CLVS1* podocytes, we evaluated the internalization of fluorescently labeled transferrin (clathrin-mediated endocytosis), 70,000 MW dextran (macropinocytosis), and albumin molecules (caveolae-mediated endocytosis). (**A** and **B**) Quantification of the number of internalized transferrin molecules per cell revealed a decrease in clathrin-mediated endocytosis in *CLVS1*-KO and homozygous H310Y-KI podocytes compared with controls that was unaffected by pretreatment with 1 μM dexamethasone. (*n* > 30 for each group, *P* < 0.0001, 1-way ANOVA.) (**C** and **D**) Macropinocytosis, as measured by the internalization of fluorescently labeled 70,000 MW dextran molecules, was unaffected by *CLVS1* KO or H310Y KI (*P* = 0.9572 and *P* = 0.9604 respectively, 1-way ANOVA). However, treatment with dexamethasone did significantly increase macropinocytosis in all cell lines compared with vehicle-treated controls (*P* < 0.001 for all). (**E** and **F**) Caveolae-mediated endocytosis was comparable among *CLVS1*-KO, homozygous H310Y-KI podocytes, and controls when internalized albumin molecules were quantified, and this was unaffected by treatment with dexamethasone (*n* > 30 for each group, *P* = 0.993 and *P* = 0.1844 respectively, 1-way ANOVA). Error bars depict SEM in graphs. Scale bars = 12 μm. Each dot in the graphs represents the mean number of molecules per podocyte for a single well.

Dextran molecules of this size are internalized through multiple modes of endocytosis, including macropinocytosis, caveolae-mediated endocytosis, and CME. To identify the specific forms of endocytosis affected by deficiencies in *CLVS1*, we used automated cell imaging to quantify the internalization of fluorescently labeled transferrin and albumin molecules in cultured human podocytes. Transferrin molecules are internalized through CME while albumin molecules undergo caveolae-mediated endocytosis. As can be seen in Figure 5, *CLVS1*-KO and homozygous H310Y-KI podocytes displayed decreased CME that was unresponsive to corticosteroid treatment. Caveolae-mediated endocytosis, however, was unaffected by the loss of *CLVS1* or the H310Y variant. While macropinocytosis was also unaffected in *CLVS1*-KO and H310Y-KI cell lines, the treatment with steroids did provide a significant increase in macropinocytosis in all podocyte cell lines (*P* < 0.0001 between treatment groups for each cell line). These data confirm the suspected role for clavesin-1 in CME and suggest that CME-independent corticosteroid-responsive mechanisms, including macropinocytosis, likely contribute to the phenotype rescue in *CLVS1*-KO and H310Y-KI podocytes.

### The CLVS1 p.H310Y variant disrupts CLVS1 ligand binding.

**Figure 6:**
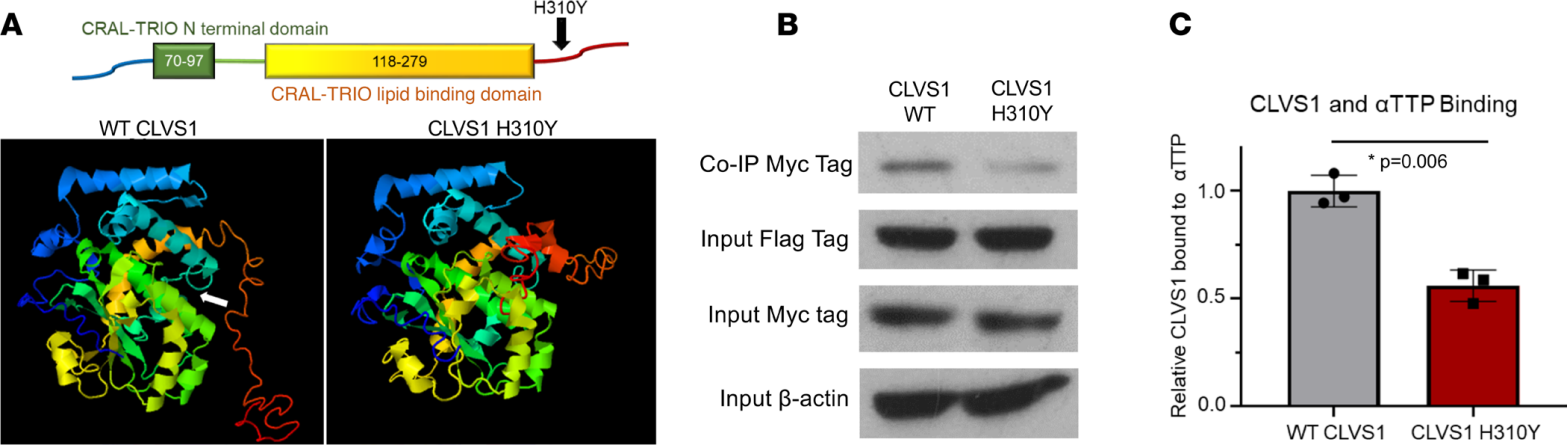
The *CLVS1* p.H310Y variant decreases clavesin-1 ligand binding to α-tocopherol transport protein. (**A**) The p.H310Y change is predicted to cause major structural alterations to the C-terminus of clavesin-1 and interfere with α-tocopherol–binding domain (white arrow). (**B** and **C**) Coimmunoprecipitation studies revealed a decrease in Myc-tagged clavesin-1 bound to the immunoprecipitated Flag-tagged αTTP when both are expressed at equivalent levels in HEK293 cells (*n* = 3, *P* = 0.006, 2-tailed *t* test).

Three-dimensional predictive modeling of the wild-type clavesin-1 protein and the p.H310Y variant revealed that this mutation will likely produce a substantial structural change to the C-terminus of the protein that will affect ligand binding (Figure 6A). While the C-terminus of clavesin-1 is important for clathrin binding and is likely to be severely impacted, the ligand predicted to be most affected by this structural alteration is the α-tocopherol transfer protein (αTTP) bound to α-tocopherol (Supplemental Figure 6). The main functional component of vitamin E present in humans, α-tocopherol functions as an antioxidant due to its free radical–scavenging abilities (28–31). Treatment with α-tocopherol has been shown to have a protective effect on podocytes in mouse models of glomerular disease (32–37). We confirmed the p.H310Y-mediated disruption of clavesin-1 binding to αTTP through coimmunoprecipitation studies in HEK293 cells transfected with Flag-tagged αTTP and either Myc-tagged wild-type or p.H310Y *CLVS1* constructs in the presence of excess α-tocopherol (Figure 6, B and C, and Supplemental Figure 7).

### CLVS1-KO and H310Y-KI podocytes generate increased ROS.

**Figure 7:**
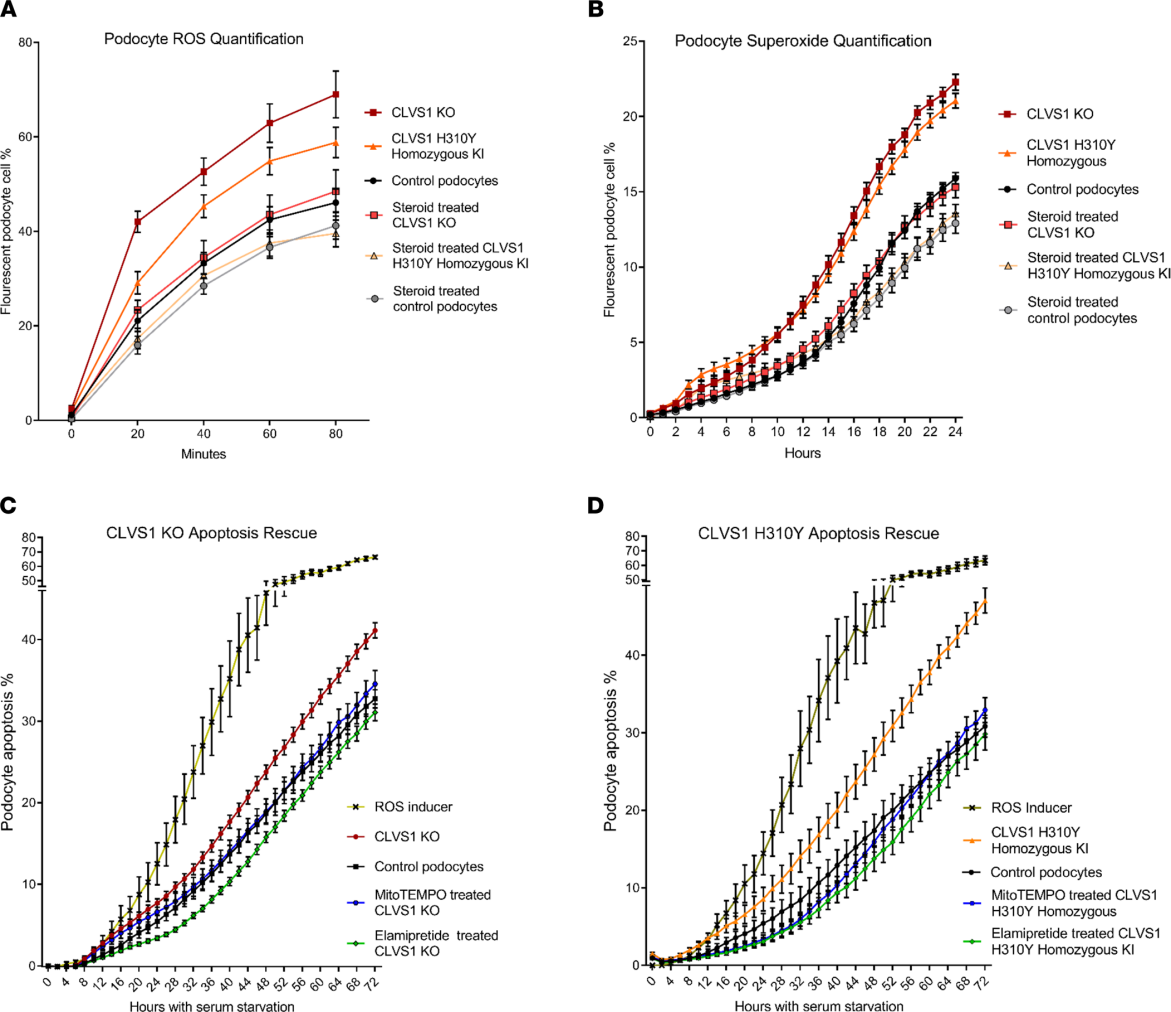
Defective ROS regulation contributes to decreased viability in *CLVS1* KO and H310Y KI podocytes. (**A** and **B**) Automated live-cell imaging and quantification of ROS levels in *CLVS1*-KO podocytes as well as controls using a fluorescent reporter of multiple ROS types, including hydrogen peroxide, peroxynitrite, and hydroxl radicals (**A**) (*n* > 20 for each group, *P* < 0.0001 for all time points for KO, *P* < 0.05 for all time points, and after 20 minutes for KI, 2-way ANOVA), as well as a second independent fluorescent reporter that detects superoxide generation (**B**) (*n* > 20 for each group, *P* < 0.001 for all time points after 10 hours, 2-way ANOVA), revealed an increase in ROS and superoxide accumulation in *CLVS1*-KO and homozygous KI podocytes that could be rescued with pretreatment with 1 μM dexamethasone. (**C** and **D**) The increased susceptibility to apoptosis in *CLVS1*-KO and homozygous KI podocytes (*P* < 0.05 for all time points after 36 hours) could be rescued by treatment with a superoxide scavenger or an ROS inhibitor (5 nM MitoTEMPO and 500 nM elamipretide) (*n* > 10 for all conditions, 2-way ANOVA). The ROS inducer pyocyanin (300 μM) and ROS inhibitor *N*-acetyl-l-cysteine (5 mM) were used as positive and negative controls, respectively, for all experiments. Error bars depict SEM.

To determine if the altered intracellular trafficking of a key antioxidant like α-tocopherol can affect podocyte oxidative stress regulation, we examined the accumulation of ROS in *CLVS1*-KO and H310Y-KI podocytes. Using automated live-cell imaging and fluorescent reporters of multiple ROS types, we quantified podocyte ROS generation with a reporter that detects hydrogen peroxide, peroxynitrite, and hydroxl radicals (Figure 7A) as well as a separate reporter for superoxide generation (Figure 7B). *CLVS1*-KO and homozygous H310Y-KI podocytes both displayed increased levels of ROS accumulation that could be restored to control podocyte levels with corticosteroid treatment.

Furthermore, to investigate the potential contribution of these increased ROS levels to deficits in *CLVS1* podocyte viability, we treated *CLVS1*-KO podocytes with compounds targeting decreased ROS accumulation and examined their effects on apoptosis. Treatment with elamipretide, an inhibitor of mitochondrial ROS generation, and MitoTEMPO, a mitochondrially targeted superoxide scavenger, was sufficient to restore podocyte viability in *CLVS1*-KO podocytes (Figure 7, C and D). Taken together, these data suggest that oxidative stress regulation may be compromised in patients carrying the *CLVS1* p.H310Y variant and that increased ROS accumulation is likely a contributing factor in *CLVS1*-mediated NS. Moreover, drugs targeting ROS accumulation and oxidative stress represent possible therapeutic options for these patients.

## Discussion

In the present study, we showed that *CLVS1* encodes an essential component of podocyte CME and that a rare homozygous variant in this gene (p.H310Y) is a potential cause of corticosteroid-responsive NS. The p.H310Y variant was deleterious to clavesin-1 function, resulting in corticosteroid-responsive phenotypes in human podocytes, including increased ROS accumulation and decreased viability. While the possible contributions of the other 3 variants found in this study cannot be completely discounted, the accumulated genetic and functional data in this study indicate that *CLVS1* is a candidate gene for autosomal-recessive SSNS.

The loss of *CLVS1* resulted in defective podocyte endocytosis, suggesting that proper regulation of these vesicle pathways is critical to maintenance of the GFB. This vital role for podocyte endocytosis in NS is supported by recent findings by other investigators. Both clathrin-dependent and clathrin-independent endocytosis serve critical roles in the regulation of podocyte-associated slit diaphragm proteins, including nephrin (15–18). Mutations in endocytosis-related proteins *GAPVD1*, *ANKFY1*, and *TBC1D8B* have now been identified as causes of monogenic NS (19, 20). Furthermore, disruption of key endocytosis-related genes in murine models have been shown to be sufficient to induce proteinuria (17, 37, 38). However, the molecular mechanisms responsible for disruptions in GFB integrity resulting from defective podocyte endocytosis have yet to be fully elucidated.

In addition to defects in podocyte endocytosis, *CLVS1* p.H310Y is also predicted to disrupt the ligand binding site for αTTP bound to α-tocopherol, which is a mitochondrial antioxidant that contributes to the regulation of lipid peroxidation and oxidative stress. The importance of mitochondrial antioxidants to glomerular disease has become an area of intense research as it has become increasingly apparent that increased oxidative stress is a common feature of chronic and progressive chronic kidney disease (39–42). Mutations in genes related to coenzyme Q_10_, another mitochondrially associated antioxidant, including *PDSS1*, *PDSS2*, *COQ2*, *COQ6*, and *COQ8B/ADCK4*, are associated with the development of glomerular disease (43–47). While mutations in αTTP are known to produce vitamin E deficiency associated with sensory ataxia in humans, its effects on kidney function are unclear (48). However, the serum levels of vitamin E have been shown to fluctuate significantly between disease and remission states in patients with NS and are reported to be reduced in those with a frequently relapsing disease course (49–57). Moreover, vitamin E deficiency can lead to abnormal kidney development in murine models of disease (58). Treatment with α-tocopherol has been reported to reduce proteinuria in multiple independent mouse models of glomerular and podocyte injury and has shown potential to improve proteinuria in patients with NS as a supplemental treatment (32, 34–36, 59, 60). The discovery of a pathogenic mutation in patients with SSNS that has the potential to alter vesicle trafficking of α-tocopherol in podocytes provides further evidence of the importance of podocyte ROS regulation and mitochondrial performance for maintaining the integrity of the GFB.

We have demonstrated that dexamethasone treatment can rescue the decreased podocyte endocytosis and viability phenotypes that result from the loss of *CLVS1* expression and the homozygous H310Y variant. It is currently unknown if this restored podocyte viability is a product of the corticosteroid-induced increase in podocyte macropinocytosis to compensate for deficits in CME, or if there are additional cellular processes affected by dexamethasone treatment that contribute to the phenotype rescue. In addition to the potential endocytosis-related contributions to podocyte viability, dexamethasone may also directly affect podocyte ROS regulation, as glucocorticoids have been shown to increase antioxidant enzyme activities in rat models of glomerular disease (61). While the mechanisms promoting this improved ROS processing have yet to be fully elucidated, our data demonstrate that increasing antioxidant activity and ROS scavenging can help restore the deficits in cell viability due to *CLVS1*.

**Figure 8:**
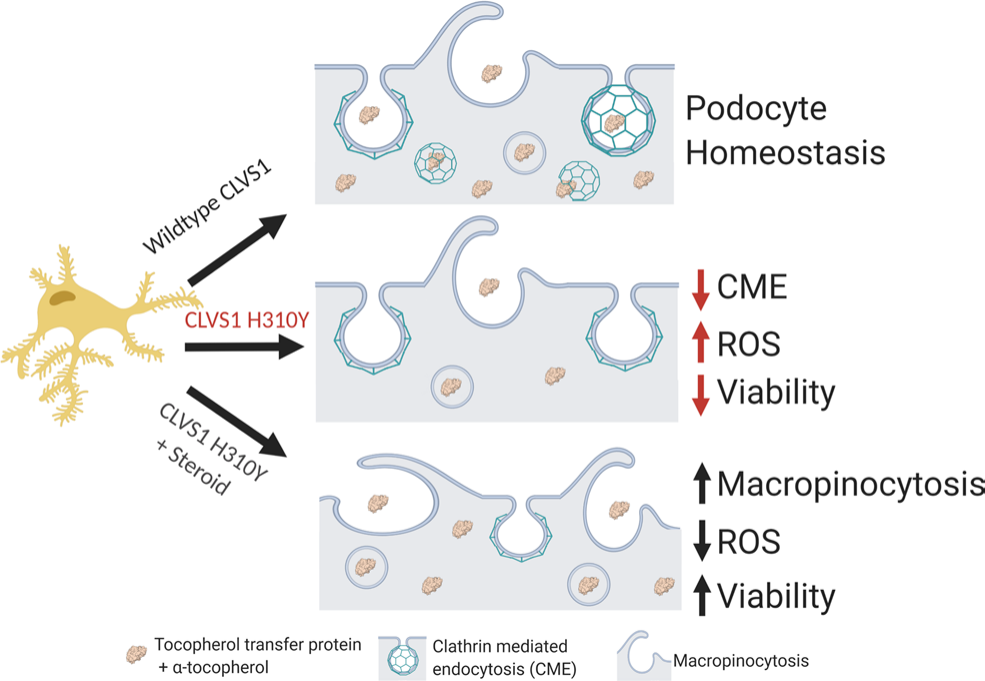
Summary figure. Based on the data acquired in this study, we posit that *CLVS1* function is required for CME in podocytes and disruptions in this process lead to disruption of GFB resulting in glomerular disease. The p.H310Y variant reduces the CME of key podocyte molecules, such as α-tocopherol, whose absence drives increased ROS and reduces viability. This pathological condition can be improved by the increased macropinocytosis and antioxidant enzyme activity in podocytes caused by corticosteroid treatment.

Based on the analysis of *CLVS1* p.H310Y, we posit that this variant disrupts the CME of key podocyte molecules, including α-tocopherol, leading to increased ROS accumulation and decreased podocyte viability (Figure 8). This reduction of podocyte health is at least partially restored by corticosteroid treatment due to an increase in macropinocytosis and ROS processing.

Limitations of our findings include the uncertainty of the contribution of the other 3 variants found in both affected and unaffected siblings to disease etiology and pathogenesis. In addition, it should be noted that the ancestry of the family in the present report (North African) is underrepresented in gnomAD; however, it is reassuring that we did not find any individual with the homozygous *CLVS1* H310Y variant in over 250,000 global exomes.

In conclusion, the discovery of another monogenic cause of SSNS that directly affects podocyte viability adds to the growing evidence that suggests that despite the major role of adaptive immunity in the pathogenesis of SSNS, primary or secondary disruption of podocyte cellular dynamics is a key component of NS pathogenesis regardless of the pattern of response to corticosteroids and other immunomodulatory treatment. Additionally, these data highlight the importance of endocytosis and ROS regulation to podocyte viability and provide insight into the therapeutic effects of corticosteroids on podocyte cellular dynamics. Continued evaluation of podocytes in the context of specific pathogenic variants like *CLVS1* p.H310Y will be critical to improving our understanding of disease pathogenesis and the development of new targeted therapies.

## Methods

### Next-generation sequencing.

Next-generation sequencing was performed at the Duke Center for Genomic & Computational Biology and GENEWIZ. We are unable to deposit the next-generation sequencing data used in this article into a public database because the families have the potential to be identified using the data. However, sequencing data are available on request. Briefly, genomic DNA samples were assessed for purity, quantity, and quality by using the NanoDrop 2000 Spectrophotometer (Thermo Fisher Scientific), Qubit 2.0 Fluorometer and Qubit dsDNA HS Assay Kit (Thermo Fisher Scientific), and agarose gel electrophoresis. Library construction was then performed using Illumina’s TruSeq DNA PCR-free library preparation kit following the manufacturer’s protocol. Genomic DNA was fragmented by acoustic shearing with a Covaris S220 instrument. Sheared DNA was then end-repaired and A-tailed, followed by adapter ligation. Final libraries were analyzed on the Agilent TapeStation for library sizing and quantified using the Qubit dsDNA HS Assay Kit and by quantitative PCR using the KAPA Library Quantification Kit. DNA libraries were sequenced using Illumina platforms to generate at least 120 Gb of raw data per sample with a 2 × 150 bp paired-end sequencing configuration. Whole-exome sequencing data were processed using the TrimGalore tool kit, which employs Cutadapt to trim low-quality bases and Illumina sequencing adapters from the 3′ end of the reads (62). Reads were aligned to the b37 version of the human genome with the Burrows-Wheeler Aligner (BWA) algorithm (63). PCR duplicates were flagged using the PICARD Tools software suite. Alignment processing and variant calling were performed using the Gene Analysis Toolkit (GATK), following the Broad Institute’s Best Practices Workflow (64, 65). Ensembl Variant Predictor (version 95) was used to annotate variant consequences and allele frequencies from gnomAD (exomes and genomes release 2.1) as well as the Exome Sequencing Project (ESP6500) (23, 66, 67). Once annotated, variants were filtered for quality using GATK’s Variant Score Quality Recalibration workflow when able; otherwise, the Broad’s recommended “hard-filtering” strategy was used. We retained variants at sites not in gnomAD or having an allele frequency less than 0.05 (in any population).

### Variant calling and annotation.

DNA-Seq data were processed using fastp1 to trim low-quality bases and Illumina sequencing adapters from the 3′ end of reads (68). Reads were then aligned to the GRCh37 version of the human genome with the BWA2 algorithm (63). PCR duplicates were flagged using the PICARD Tools software suite (69). Alignment processing and variant calling were performed using the GATK4 following the Broad Institute’s Best Practices Workflow (64, 65). Functional consequences and genotype provenances of variants were annotated using Ensembl Variant Predictor (66). Following annotation, variants meeting the following criteria were selected for further analysis: having a status of PASS following GATK’s Variant Quality Score Recalibration, residing in a coding region, and having an allele frequency of less than 5% in at least 1 population of gnomAD (22). Second level filtering to identify disease-causing variants was as shown in Supplemental Figure 1. Variants of interest were confirmed by Sanger sequencing.

### Homozygosity mapping.

Kinship between individuals was estimated using the plink2 software applying the KING-robust estimator to the whole-exome data (70, 71). ROH were estimated using the hidden Markov model implemented in the BCFtools software module RoH (72). The human recombination map was used, and only variants with FILTER = “PASS” and that were not indels were used in the analysis. Only ROH exceeding 1 Mb in length were considered. ROH after filters (present in SSNS patients and absent in unaffected individuals) were merged if the gap between region pairs was less than 1 Mb.

### Three-dimensional in silico protein modeling.

For clavesin-1 modeling, I-TASSER generated a large ensemble of structural conformations, called decoys, based on the amino acid composition. To select the final models, I-TASSER uses the SPICKER program to cluster all the decoys based on the pairwise structure similarity and reports up to 5 models that correspond to the 5 largest structure clusters. The confidence of each model is quantitatively measured using a C-score that is calculated based on the significance of threading template alignments and the convergence parameters of the structure assembly simulations (73–75). For clavesin-1 ligand binding predictions, analysis was performed by COFACTOR and COACH based on the I-TASSER structure prediction. COFACTOR deduces protein functions using structure comparison and protein-protein networks, and COACH is a meta-server approach that combines multiple function annotation results (on ligand binding sites) from the COFACTOR, TM-SITE, and S-SITE programs (76–79). Molecular graphics generation and analyses of PDB files created in the I-TASSER software were performed with UCSF ChimeraX, developed by the Resource for Biocomputing, Visualization, and Informatics at the UCSF, with support from NIH R01-GM129325 and the Office of Cyber Infrastructure and Computational Biology, National Institute of Allergy and Infectious Diseases, NIH.

### Zebrafish analysis.

All studies performed in zebrafish were approved by the Duke University Institutional Animal Care and Use Committee. We designed a translation blocking (GGCCTGCCTGTAAATGAGTCATTGT) as well as a splice blocking MO (AAAATCCAATAGCTTCCTACCTGCT) targeting the exon1/intron1 splice site of clvs2 (Ensembl ID: ENSDART00000075112.6; Gene Tools). To determine MO efficiency, we injected 8 ng of each MO or a control MO (Gene Tools) into wild-type zebrafish embryos (ZDR strain) at the 1- to 4-cell stage (1 nL/embryo) and harvested them at 2 days postfertilization (dpf) for RNA extraction (splice blocking MO) and at 3 days for protein extraction (translation blocking MO). For analysis of splice blocking MO efficiency, we used the Qiashedder and RNeasy Mini Kit (Qiagen) to extract RNA following the manufacturer’s protocol and Promega Reverse Transcription kit to perform RT-PCR, then amplified the clvs2 targeted region using custom primers (For-CTCCTGGCCCAATACTTTGA and Rev-TCCGGGTCTTCTATCATTGC). To assess the efficiency of the translation blocking MO, we processed the embryos as previously described (80). Briefly, 50 embryos were washed and dechorionated with pronase before the yolks were removed in Ringer’s solution containing PMSF and proteinase inhibitors. The remaining tissue was homogenized in 100 μL of 2× SDS and boiled for 5 minutes before performing immunoblotting as described below. We assessed the phenotypic effects of progressive doses of MO (3 ng, 5 ng, and 8 ng) and subsequently used 5 ng MO for the translation blocking morpholino and 8 ng for the splice blocking MO studies. For mRNA rescue, we obtained custom oligos with the wild-type zebrafish clvs2 ORF, wild-type human *CLVS1* ORF, and human *CLVS1* p.H310Y ORF (GenScript). All constructs were created in a pcDNA3.1 backbone and were sequence confirmed. To perform in vitro transcription, we linearized plasmids with NotI, then generated capped mRNA using the mMessage mMachine T7 Ultra transcription kit (Thermo Fisher Scientific). We injected 150 pg mRNA for all embryo injections. Live bright-field images were acquired on 4 dpf larvae anesthetized with tricaine using a Nikon AZ100 microscope with a Digital Sight color camera and NIS Elements software.

### Zebrafish GFB evaluation.

To examine the effects of clvs2 KD on the maintenance of GFB integrity, we took advantage of an established fish model for proteinuria detection, Tg(lfabp:VDBP-GFP). This line contains a fluorescently labeled vitamin D molecule, which is excreted when there are defects in GFB integrity. We injected *clvs2* translation blocking MOs and control MOs as described above into 1- or 2-cell embryos resulting from breeding wild-type fish to the GFP reporter line. Embryos were raised until 3 dpf and then transferred to a 24-well plate in groups of 3 with 500 μL of embryo water for 72 hours. Water was then collected and analyzed using a GFP ELISA kit (Boster Bio) according to the manufacturer’s protocol.

### Zebrafish electron microscopy.

Zebrafish embryos at 5 dpf were fixed in 2.5% glutaraldehyde in 0.1 M sodium cacodylate buffer. We washed the specimen in 0.1 M sodium cacodylate buffer, with 3 media changes for 15 minutes each. Samples were postfixed in 1.0% OsO_4_ in 0.10 M sodium cacodylate buffer for 1 hour on a rotator (VWR) and washed in 3 changes of working buffer, 15 minutes each change. Samples were then placed into en bloc stain (1% uranyl acetate) for 2 hours at room temperature and dehydrated in a series of ascending acetone concentrations (50%, 75%, 95%, 100% 3 times) for 10 minutes each. Specimens were placed in a 50/50 mixture of EPON/ACETONE overnight on a rotator before being replaced with 100% epoxy resin (EPON) for at least 2 hours at room temperature on a rotator. There were 2 more changes of 100% epoxy and incubation for at least another 2 hours at room temperature on a rotator. Samples were then embedded in Beem capsules for 48 hours at 60°C and were ultrathin sectioned (60–70 nm) on a Reichert Ultracut E ultramicrotome. Grids were stained with 2% uranyl acetate in 50% ethanol (EtOH) for 30 minutes and SATO’s lead stain for 1 minute and imaged on a JEOL 2100plus electron microscope. Images were evaluated by a pathologist blinded to treatment groups.

### Human podocyte cell lines.

Conditionally immortalized human podocytes (courtesy of Jefferey Kopp, NIH, Bethesda, Maryland, USA) were grown, maintained, and differentiated as described previously (81, 82). To create the *CLVS1*-KD lines, we used lentiviral particles containing shRNA against *CLVS1* (MilliporeSigma). Lentiviral podocyte control lines were made using lentiviral particles containing nontargeted shRNA, and knockdown was confirmed through Western blot analysis (75).

CRISPR/Cas9-mediated *CLVS1* KO in the conditionally immortalized human podocytes was performed by the Duke Functional Genomics Core. Paired sgRNAs targeting exon 3 of *CLVS1* were designed using ChopChop (sgRNA 1, GGAAGTCCTAATCGAAGATC; sgRNA 2, ATGACAGCAGGATGGCACGA) (83). The sgRNAs were ordered as modified synthetic sgRNAs from Synthego. A total of 1 × 10^5^ podocytes were electroporated with 15 pmol sgRNA complexed with 15 pmol TrueCut Cas9 protein v2 (Thermo Fisher Scientific) using a Neon system (Thermo Fisher Scientific) with the following settings: 1300 V, 20 ms, 2 pulses. Cells were recovered on collagen-coated, 6-well plates for a period of a week, followed by plating at limiting dilution onto collagen-coated 96-well plates. Clones were expanded for several weeks and screened using PCR sequencing to confirm out-of-frame insertions or deletions. Selected clones were also sequenced by TA cloning and Sanger sequencing to further confirm the presence of frameshifting indels (Supplemental Figure 8). The lines were evaluated independently before being combined in the final analysis for clarity. Unmodified human podocytes were used as control comparisons for the CRISPR-KO lines.

KI podocytes were created to introduce the c. 928T, p. H310Y mutation into the coding sequence of *CLVS1*. A total of 1 × 10^5^ podocytes were electroporated with 15 pmol sgRNA complexed with 15 pmol TrueCut Cas9 protein v2 and 10 pmol of single-stranded oligonucleotide donor using a Neon system with the following settings: 1300 V, 20 ms, 2 pulses. The sgRNA (GAAGCATACGTCCTCGAATC) was ordered as modified synthetic sgRNA from Synthego. The oligo donor sequence was designed with the desired mutation as well as 2 additional silent mutations to prevent recleavage by Cas9 after homology-directed repair. The donor sequence is shown below and was ordered as an ultramer with 2 phosphorothioate bonds at each end from Integrated DNA Technologies. After electroporation, cells were recovered on collagen-coated, 6-well plates for a period of a week, followed by plating at limiting dilution onto collagen-coated, 96-well plates. Clones were expanded for several weeks and screened by PCR using a primer pair that specifically detects the KI sequence. Positive clones were then subjected to PCR sequencing to confirm the presence of the desired genomic alterations (Supplemental Figure 9) (donor: gcgatgaaaatgactatactcacacatcctataatgcaatgcacgtgaagtatacttcttcgaatctggagagagaatgctcacccaagctgatgaaaag).

### HEK293 cell transfection.

For the studies, HEK293 cells (HEK293T/17, ATCC) were grown in Dulbecco’s modified Eagle medium supplemented with 10% fetal calf serum, penicillin (100 U/mL), and streptomycin (100 μg/mL) (all from Gibco) as previously described (84). For transfection, HEK293 cells were plated in 6-well tissue culture plates (Costar Corning) and grown to approximately 80% confluence. Cells were then cotransfected with the Flag-tagged calcineurin α-tocopherol transfer protein (TTPA) construct (GenScript) and the Myc-tagged *CLVS1* construct (wild-type or p.H310Y mutant as indicated) (GenScript) using Lipofectamine 2000 according to the manufacturer’s recommendations (Thermo Fisher Scientific).

### Coimmunoprecipitation studies.

To achieve overexpression of tagged *TTPA* and *CLVS1* constructs, we used HEK293 cells because of the relative ease of transfection. Cells were harvested approximately 68 hours after transfection with Flag-tagged calcineurin TTPA and one of the Myc-tagged *CLVS1* constructs. Coimmunoprecipitation was achieved using a Flag Immunoprecipitation Kit (MilliporeSigma) and a Myc-tag Co-IP kit (Thermo Fisher Scientific) according to the manufacturers’ protocols. Immunoblotting was performed as described below for the immunoprecipitation lysates as well as input lysates to ensure equal loading between samples.

### Automated cell apoptosis imaging.

To both visualize and quantify the apoptosis and total cell death in *CLVS1*-KO podocytes and transfected HEK293 cells, we used a Lionheart FX automated microscope from BioTek along with fluorescent apoptosis reagents. Cells were grown in 96-well plates before exposure to serum-free media containing a 1:500 dilution of NucView Caspase-3 Alexa Fluor 488 (Biotium) and a 1:2000 dilution of PI (MilliporeSigma). The NucView reagent consists of a substrate of caspase-3 that emits green fluorescence when cleaved, while PI fluoresces in late apoptotic and necrotic cells. This media also contained either 1 μM dexamethasone or an equal concentration of vehicle (EtOH). Bright-field images along with green and red fluorescent images were collected every 2 hours for 48 hours. Using automated GEN5 software from BioTek, the images were processed to remove background, and the number of fluorescent cells was quantified for each well using label-free cell counting. Wells containing full serum were used as a control to test the validity of the apoptosis readings. The experiments were repeated in quadruplicate with a total *N* of at least 16 for each cell type.

### Endocytosis assays.

To measure general endocytosis in human podocyte cells and HEK293 cells, we used a 10,000 MW pHrodo green fluorescently labeled dextran (Thermo Fisher Scientific). Cells were plated in 96-well pates. Podocyte plates were collagen coated. Cells were grown to 80% confluence, then washed twice with PBS, and then exposed to a 1:75 dilution of pHrodo in imaging solution for 25 minutes at 37°C, before it was removed and replaced with fresh imaging media and immediately imaged with the Lionheart FX automated microscope (BioTek). Fluorescently labeled molecules CF-Transferrin (Biotium), CF-Bovine Serum Albumin (Biotium), and Texas Red–Dextran 70,000 MW (Thermo Fisher Scientific) were used to examine podocyte CME, caveolae-mediated endocytosis, and macropinocytosis, respectively, according the manufacturer’s protocols. Briefly cells were exposed to serum-free media for 1 hour before adding the transferrin, albumin, and dextran reagents (1 mg/mL, 2 mg/mL, 1 mg/mL in PBS, respectively). Podocytes were incubated at 37°C in the dark for 90 minutes and then washed twice with PBS followed by imaging media and imaged with the Lionheart FX. Using automated GEN5 software from BioTek, all images were processed to remove background, and the number of fluorescent molecules per cell was quantified for each well using label-free cell counting. For dexamethasone rescue, cells were exposed to 1 μM dexamethasone for 3 hours before beginning the assay.

### ROS quantification.

To visualize and quantify the ROS accumulation within podocytes, we used the ROS-ID Total ROS Detection Kit (Enzo Biosciences) according to the manufacturer’s protocol for the quantification of ROS, including hydrogen peroxide, peroxynitrite, and hydroxl radicals. Briefly, podocytes were cultured in collagen-coated, 96-well plates. Media were replaced with ROS detection solution and imaged every 20 minutes over 1 hour using a Lionheart FX automated imaging system. Additionally, we used MitoSOX (Thermo Fisher Scientific) for detection of mitochondrial superoxide. Briefly, we used 3.5 μM of MitoSOX reagent in minimal media and imaged the cells every hour for 24 hours using the Lionheart FX imaging system. For dexamethasone rescue, cells were exposed to 1 μM dexamethasone for 3 hours before beginning the assays.

### ROS inhibition.

To examine the effects of reduced ROS levels on podocyte viability, we used 5 nM of MitoTEMPO (Cayman Chemicals), 500 nM of elamipretide (MedChemExpress), and 10 μM of α-tocopherol (MilliporeSigma). All reagents were diluted 1:1000 from EtOH stock and added to serum-free media alongside the apoptosis detection reagents.

### Mouse glomerular immunofluorescence.

We isolated mouse glomeruli from mouse kidneys by differential sieving with 180 μm, 100 μm, and 71 μm metal sieves (Retsch) and washing with PBS (Gibco 10010-023). Glomeruli were plated onto a collagen I–precoated flask (Corning 356484) and cultured for 6 days with RPMI media (Gibco 11875-093) before an outgrowth of cobblestone-like cells was observed and harvested. Cells were fixed with 4% paraformaldehyde in PBS (Electron Microscopy Sciences t15710) and washed with ice-cold PBS 3 times before permeabilization with 0.1 Triton X-100 in PBS. Cells were then washed with wash buffer (0.5% BSA, 0.05% Triton X-100 in PBS) twice and blocked with buffer containing 5% goat serum, 1% BSA, and 0.1% Triton X-100 in PBS before incubation with rabbit polyclonal CLVS1 antibody (PA5-32088 Invitrogen) and mouse monoclonal WT1 antibody (sc-7385 Santa Cruz Biotechnology) overnight at 4°C. After additional washes, secondary Alexa Fluor 488 and 568 antibodies were applied (Invitrogen A11008 and A11004, respectively) at a concentration of 1:400 for 1 hour at room temperature. Cells were then washed with wash buffer 4 times before addition of DAPI stain. Immunofluorescence imaging was performed using an EVOS FL imaging system.

### Immunoblotting.

Immunoblotting was performed using standard methods and visualized by enhanced chemiluminescence as previously described (81). Antibodies were used at the following concentrations: CLVS1 (C82727 Lifespan Biosciences and PA5-32088 Invitrogen) 1:500, β-actin (6609 Proteintech) 1:3000, Myc-tag (PA1-981 Invitrogen) 1:800, DYKDDDDK (D6W5B Cell Signaling Technology) 1:800. Uncropped Western blots are shown in Supplemental Figure 10.

### Illustrations.

The graphical abstract and Figure 8 were created with BioRender.com.

### Statistics.

Zebrafish larval batches were compared using χ^2^ tests (GraphPad Prism). One-way ANOVA followed by a Tukey’s honestly significant differences post hoc test was used to determine the differences between means for the analysis of endocytosis and immunoblotting results. Two-way ANOVA followed by a Dunnett’s multiple comparisons analysis was used to compare groups over time for automated live-cell apoptosis and ROS imaging. Statistical significance was established at *P* < 0.05. All data are represented as the mean ± SEM.

### Study approval.

All human studies were approved by the Duke University Medical Center Institutional Review Board, and written informed consent was received from participants prior to inclusion in the study. Animal experiments were approved by the Duke University Medical Center Institutional Animal Care and Use Committee.

## Author contributions

BML and RG designed the experiments and wrote the manuscript. All authors reviewed and edited the manuscript. Participant enrollment and sample acquisition were performed by MS, SE, JAK, GH, and RG. Sequencing and analysis of sequencing data were carried out by MCS, BML, and RG. BML, CCM, and GW performed the experiments. Homozygosity mapping and analysis were performed by AS, AO, and KH. Single-cell sequencing data analysis was performed by AFM. CRISPR/Cas9 gene editing in podocytes was performed by SYK. RV and BML performed the electron microscopy while SEM and DNH evaluated the images for podocyte health.

## Supplementary Material

Supplemental data

Supplemental video 1

Supplemental video 2

Supplemental video 3

## Figures and Tables

**Table 1:**
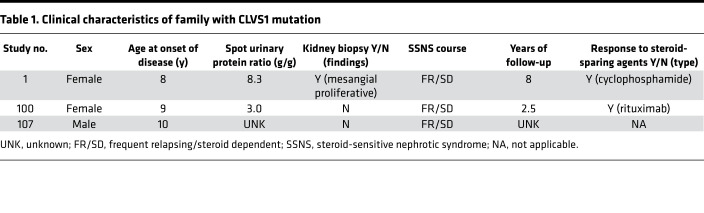
Clinical characteristics of family with CLVS1 mutation

**Table 2:**
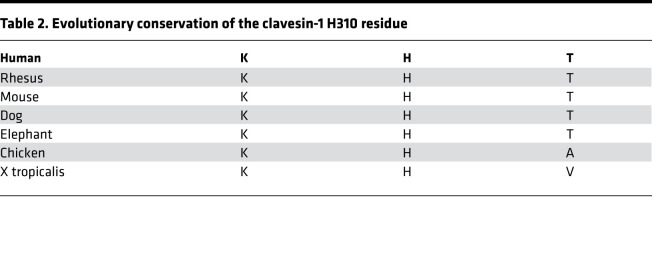
Evolutionary conservation of the clavesin-1 H310 residue

## Version 1 12/07/2021

In-Press Preview

## Version 2 01/25/2022

Electronic publication
