# Supplementary material for: Steroid-sensitive nephrotic syndrome candidate gene CLVS1 regulates podocyte oxidative stress and endocytosis
Source: JCI Insight. 2022 Jan 25;7(2):e152102. doi: 10.1172/jci.insight.152102 (PMC9018043; doi:10.1172/jci.insight.152102)
Supplement: Supplemental data [file jciinsight-7-152102-s011.pdf]

Supplementary Material

Steroid-sensitive nephrotic syndrome candidate gene CLVS1 regulates podocyte oxidative stress and endocytosis

Brandon M Lane[1], Megan Chryst-Stangl[1], Guanghong Wu[1], Mohamed Shalaby[2], Sherif El Desoky[2], Claire C Middleton[1], Kinsie Huggins[1], Amika Sood[3], Alejandro Ochoa[3], Andrew F Malone[4], Ricardo Vancini[5], Sara E Miller[5], Gentzon Hall[1,6], So Young Kim[7], David N Howell[5], Jameela A Kari[2], Rasheed Gbadegesin[1,6]

Supplementary Table 1: Homozygous Variants found in a family with hereditary SSNS and *in-silico* data

Gene (variant)	gnomAD Allele Frequency	Homozygous in gnomAD	Homozygous variant in unaffected family members?	CADD Score	SIFT	Polyphen	Mut. Taster	Conservation
***CLVS1* (H310Y)**	15 / 281974	No	No (0)	22.6	Damaging	Possibly Damaging	Disease Causing	Frog
***COL6A1* (P827L)**	6 / 267418	No	Yes (1)	27.0	Damaging	Probably Damaging	Polymorphism	Frog
***MX2* (A516T)**	14 / 282404	No	Yes (2)	27.0	Damaging	Probably Damaging	Disease Causing	Lamprey
***EML4* (D243Y)**	NA	No	Yes (1)	25.9	Damaging	Probably Damaging	Disease Causing	Lamprey

Supplementary Table 2: Summary of Homozygosity Mapping in a Consanguineous Family with Hereditary SSNS

Chromosome	Homozygosity Region*	Size (MB)	Candidate Genes in Homozygosity Regions	Known Genes in Homozygosity Regions
**1**	20,639,207 - 27,023,656	6.38	0	0
**4**	98,761,900 - 100,334,066	1.57	0	0
**8**	54,138,291 - 77,616,519	23.48	1 *(CLVS1)*	0
**10**	97,987,378 - 108,338,917	10.35	0	1 *(PAX2)* +
**21**	42,317,771 - 46,950,863	4.63	1 *(MX2)*	0

* Based on Human GRCH37/hg19
+ *PAX2* is an autosomal dominant gene

Supplementary Figure 1: Variant Filtering

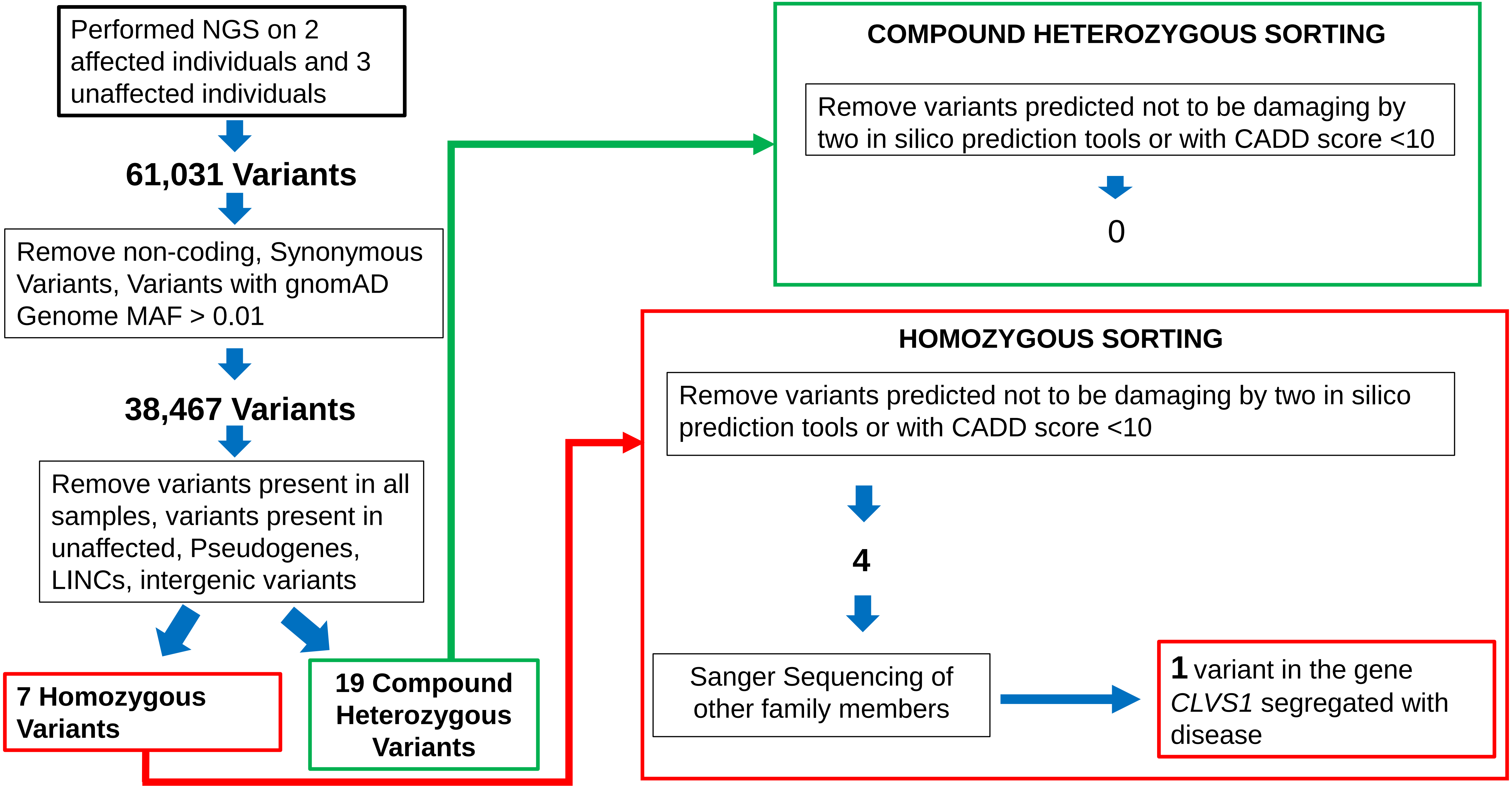

Supplementary Figure 2: *CLVS1* Expression in podocytes

***CLVS1* is expressed in podocytes**. **A)** Immunofluorescence imaging of extracted mouse glomeruli revealed co-localization of CLVS1 and WT1 proteins in podocytes (bars =100µM). **B)** *CLVS1* expression was confirmed in the extracted mouse podocytes with immunoblotting. **C)** *CLVS1* is highly expressed in human neuron cell lines (SH-SY5Y) compared to the conditionally immortalized human podocyte cell lines. **D-F**) Analysis of publicly available human kidney single cell sequencing data confirmed expression of *CLVS1* in podocytes. (26)

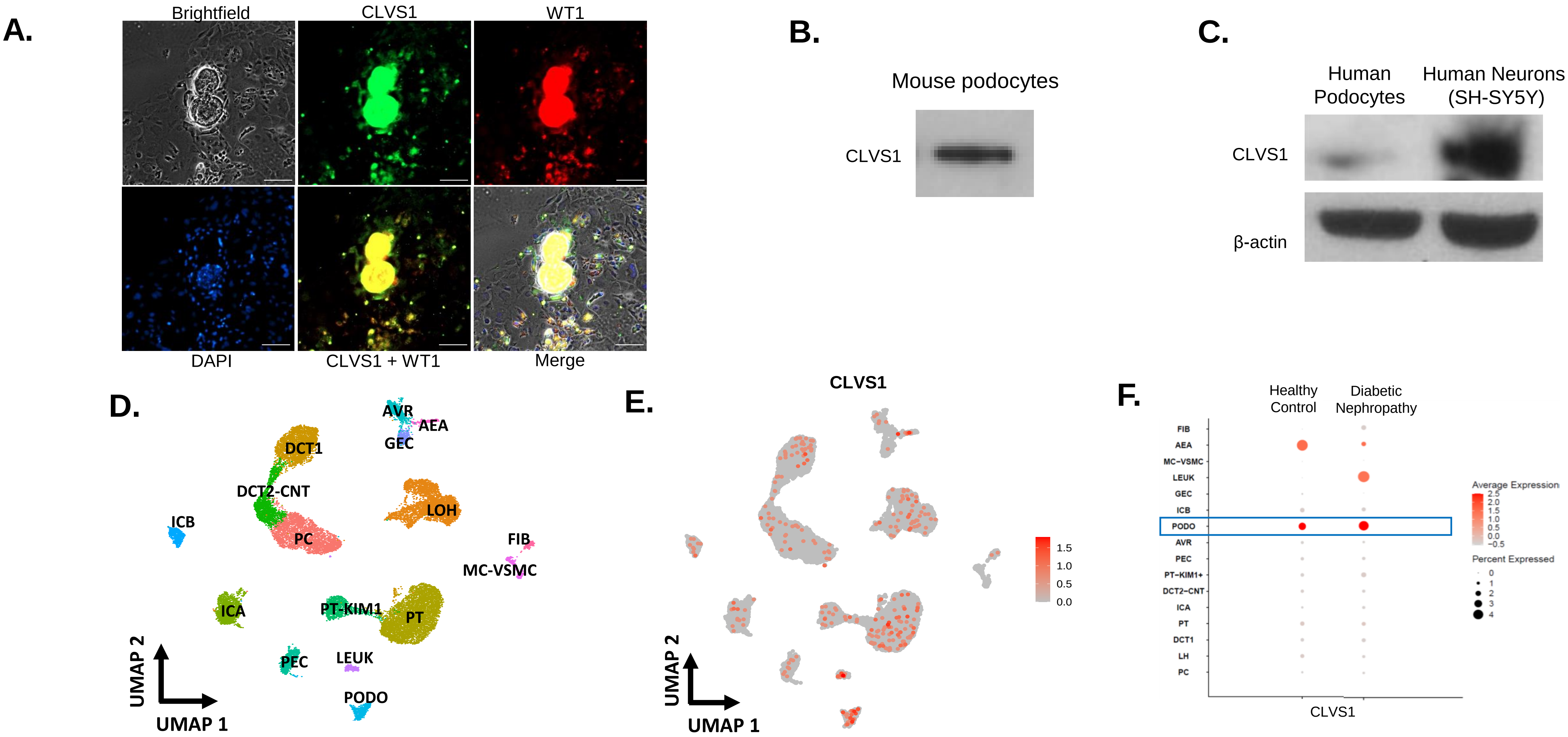

Supplementary Figure 3: *CLVS1* Late Apoptosis/Necrosis

***CLVS1* KO and H310Y KI increases total cell death in podocytes.** Quantification of Propidium revealed an increase in podocyte late apoptosis/necrosis in podocytes with *CLVS1* KO and homozygous H310Y KI podocytes compared to controls (*p<0.05 for all time points after 20 hours, N>25 for each group, two-way ANOVA) which was rescued by treatment with 1µM Dexamethasone.

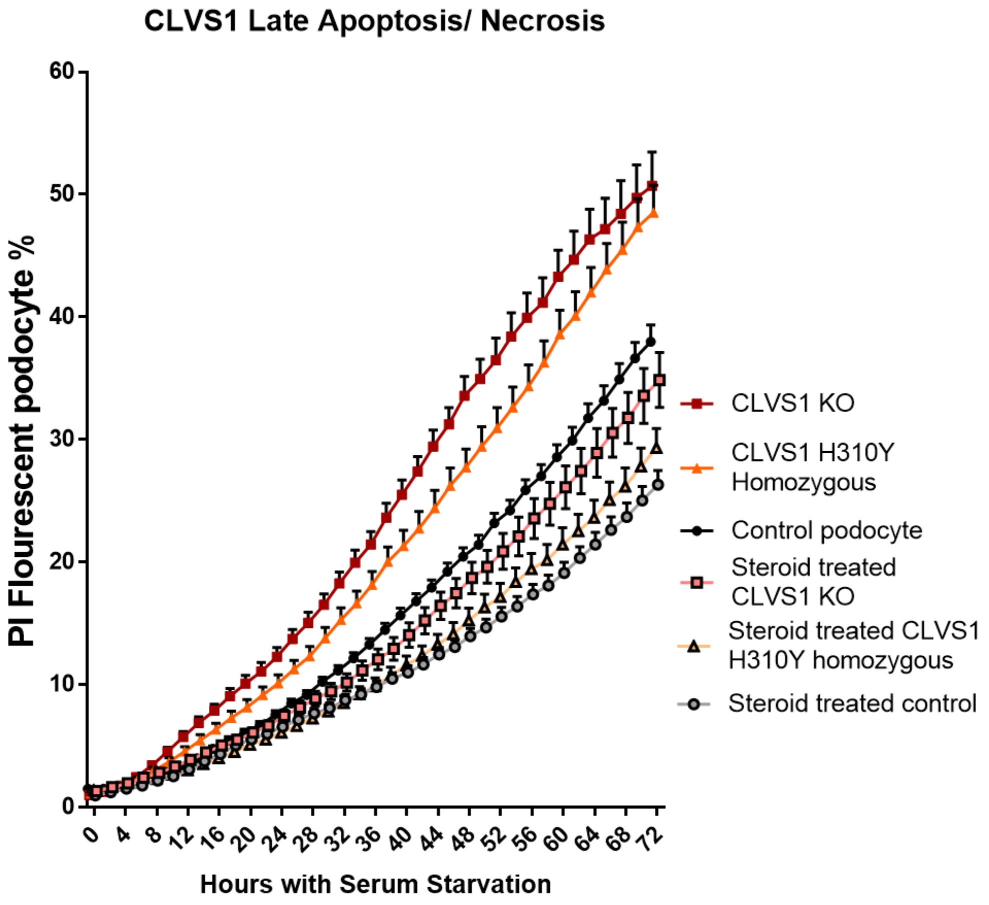

Supplementary Figure 4: *CLVS1* KD Podocyte Apoptosis and Endocytosis

***CLVS1* knockdown increases podocyte susceptibility to apoptosis and endocytosis**. **A)** Quantification of cleaved caspase 3 revealed an increase podocyte susceptibility to serum starvation induced apoptosis in podocytes with *CLVS1* knockdown (KD) compared to controls (*p<0.05 for all time points after 30 hours, N>15 for each group, two-way ANOVA) which was rescued by treatment with 1µM Dexamethasone. **B)** Similar to *CLVS1* KO podocytes, *CLVS*1 KD also decreased endocytosis of fluorescently labeled phRODO molecules (p=0.0161, n=28 each, one-way ANOVA) that could be rescued with steroid treatment (p=0.9331) .

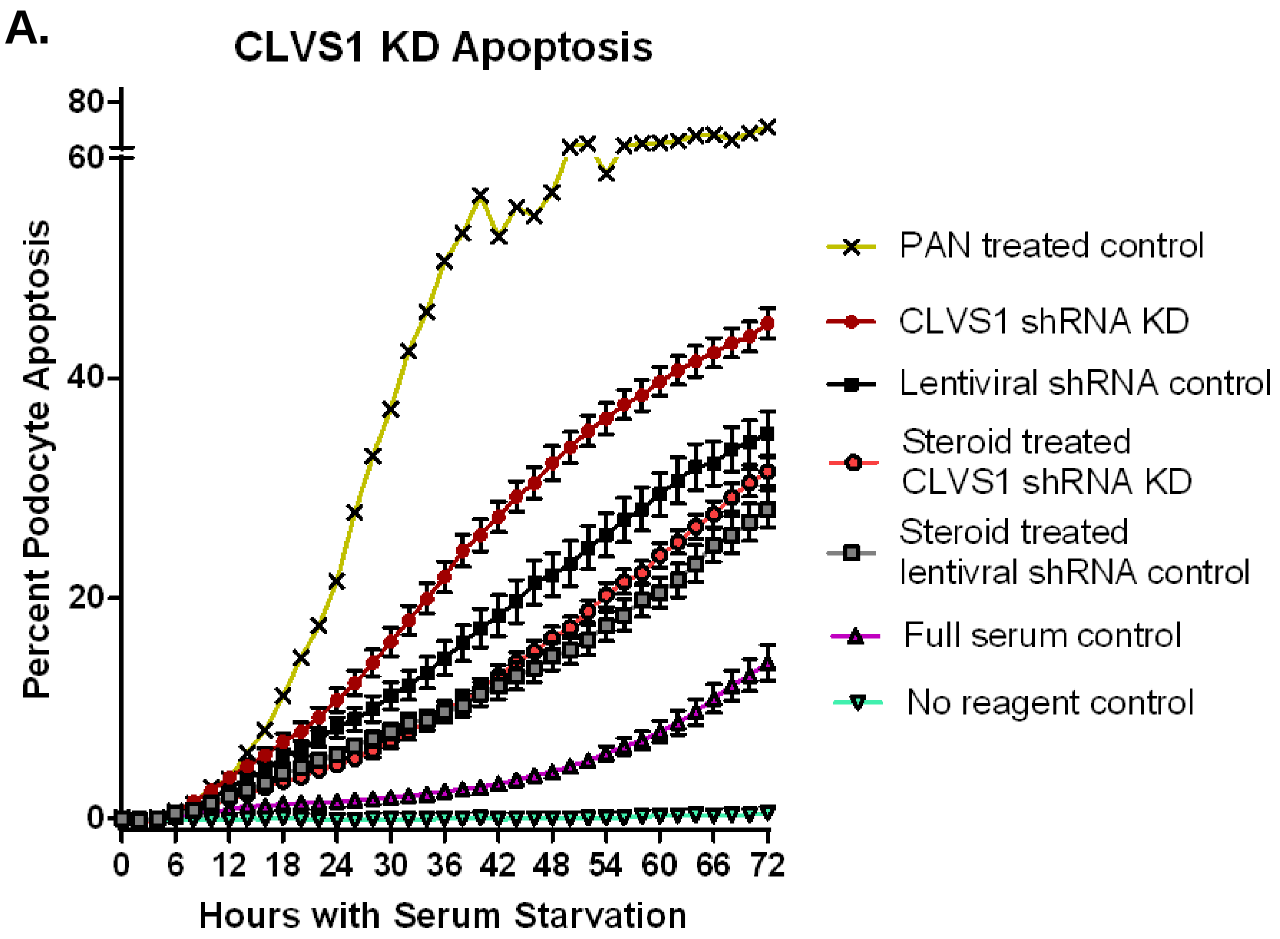

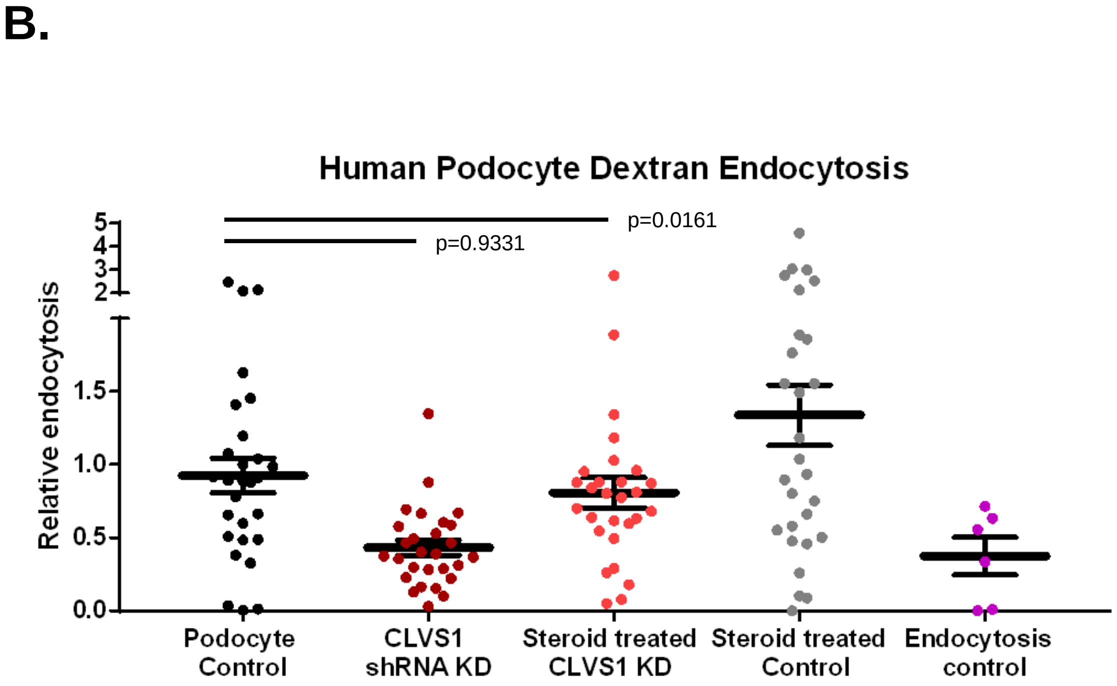

Supplementary Figure 5: HEK293 Cell CLVS1 Analysis

The *CLVS1* p.H310Y variant decreases cellular endocytosis and viability. A) Western blot showing similar levels of Myc-tagged *CLVS1* expression in WT and p.H310Y transfected HEK293 cells. **B)** Overexpression of the *CLVS1* p.H310Y variant increased susceptibility to apoptosis in HEK293 cells (n>12, p<0.05 for all time points > 14hours, two-way ANOVA) that could be rescued with 1µM Dexamethasone. (p>0.05 for all time points, two-way ANOVA) **C)** *CLVS1* p.H310Y expressing HEK293 cells displayed decreased internalization of internalized fluorescently labeled dextran molecules (pHrodo) compared to their respective controls (n=23, p=0.026, one-way ANOVA). These endocytosis deficiencies can be restored with pre-treatment with 1µM Dexamethasone (n=23, p=0.9824, one-way ANOVA).

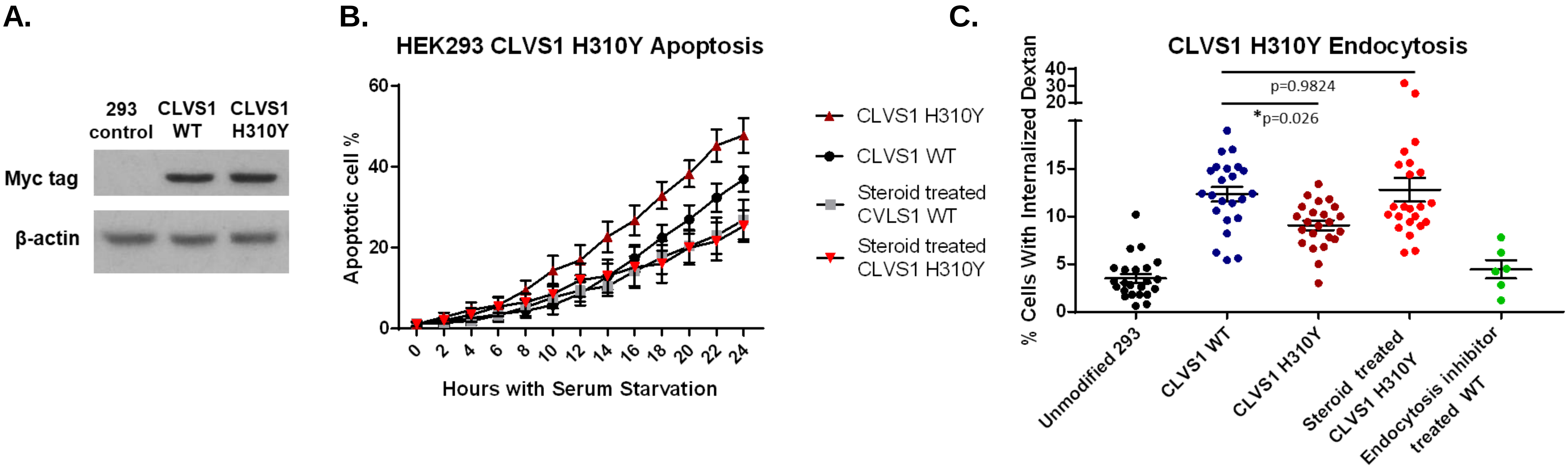

Supplementary Figure 6: Protein Binding Predictions

The *CLVS1* p.H310Y variant alters ligand binding. A-B) Protein ligand predictions and biological annotations of the target protein by COFACTOR and COACH based on the I-TASSER structure prediction revealed that one of the top clavesin-1 ligands (A), VIV, was disrupted by the H310Y mutation (B). **C)** Two views of a model of the alpha tocopherol transport protein bound to a wild type clavesin-1 molecule in the region predicted to be disrupted by the structural effects of H310Y.

A.

Predicted CLVS1 WT Ligands

Rank	C-score	Cluster size	PDB Hit	Ligand Name
1	0.32	17	3hy5A	RET
2	0.2	14	1r5lA	VIV
3	0.13	7	3w67B	3PT
4	0.05	5	3b7nA	B7N
5	0.04	3	1oizA	TRT

B.

Predicted CLVS1 H310Y Ligands

Rank	C-score	Cluster size	PDB Hit	Ligand Name
1	0.26	16	3hy5A	RET
2	0.21	15	1oizB	TRT
3	0.07	6	3w67B	3PT
4	0.05	5	3b7nA	B7N
5	0.04	3	4j7qA	B7N

C.

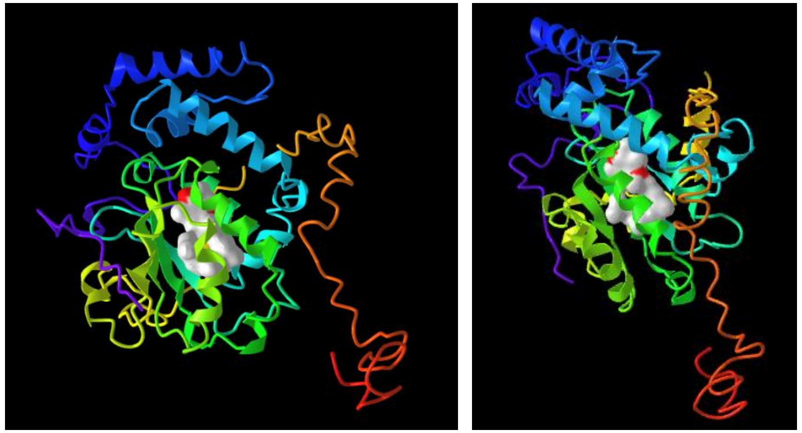

Supplementary Figure 7: Co-IP Myc Tag Pulldown

The *CLVS1* p.H310Y variant decreases binding to αTTP. Co-immunoprecipitation studies revealed a decrease in Flag-tagged αTTP bound to the immunoprecipitated Myc-tagged clavesin-1 when both are expressed at equivalent levels in HEK293 cells.

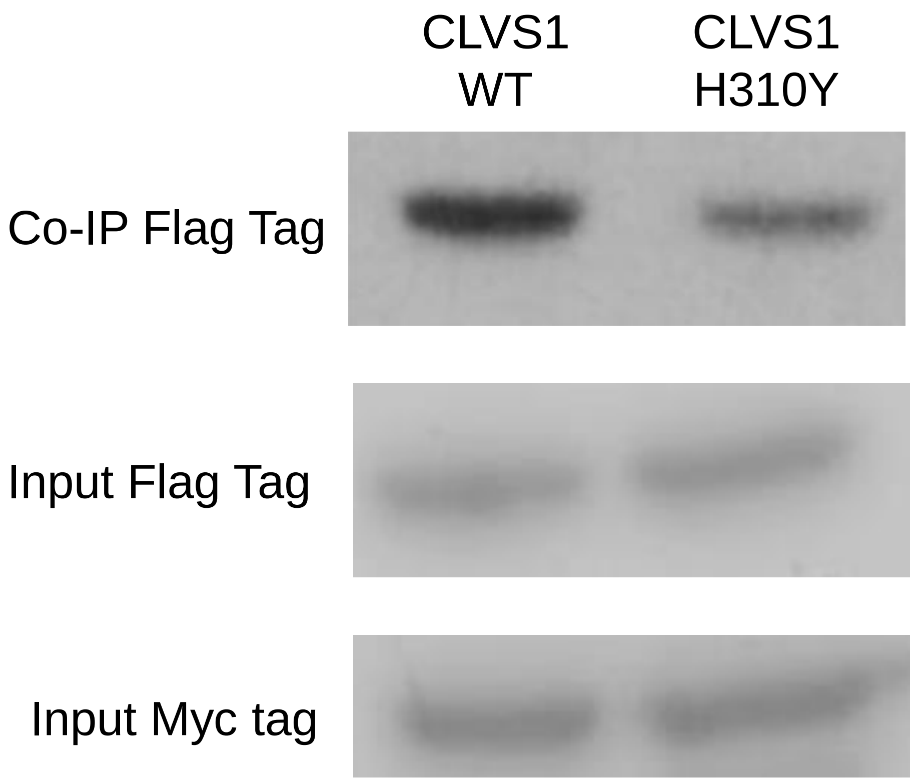

Supplementary Figure 8: *CLVS1* KO lines

***CLVS1* KO Podocyte Lines.** **A**) Schematic depicting the targeting strategy for KO line creation with primers and sgRNA as well as the deletions present in each of the two *CLVS1* KO podocyte lines that were examined in this study.

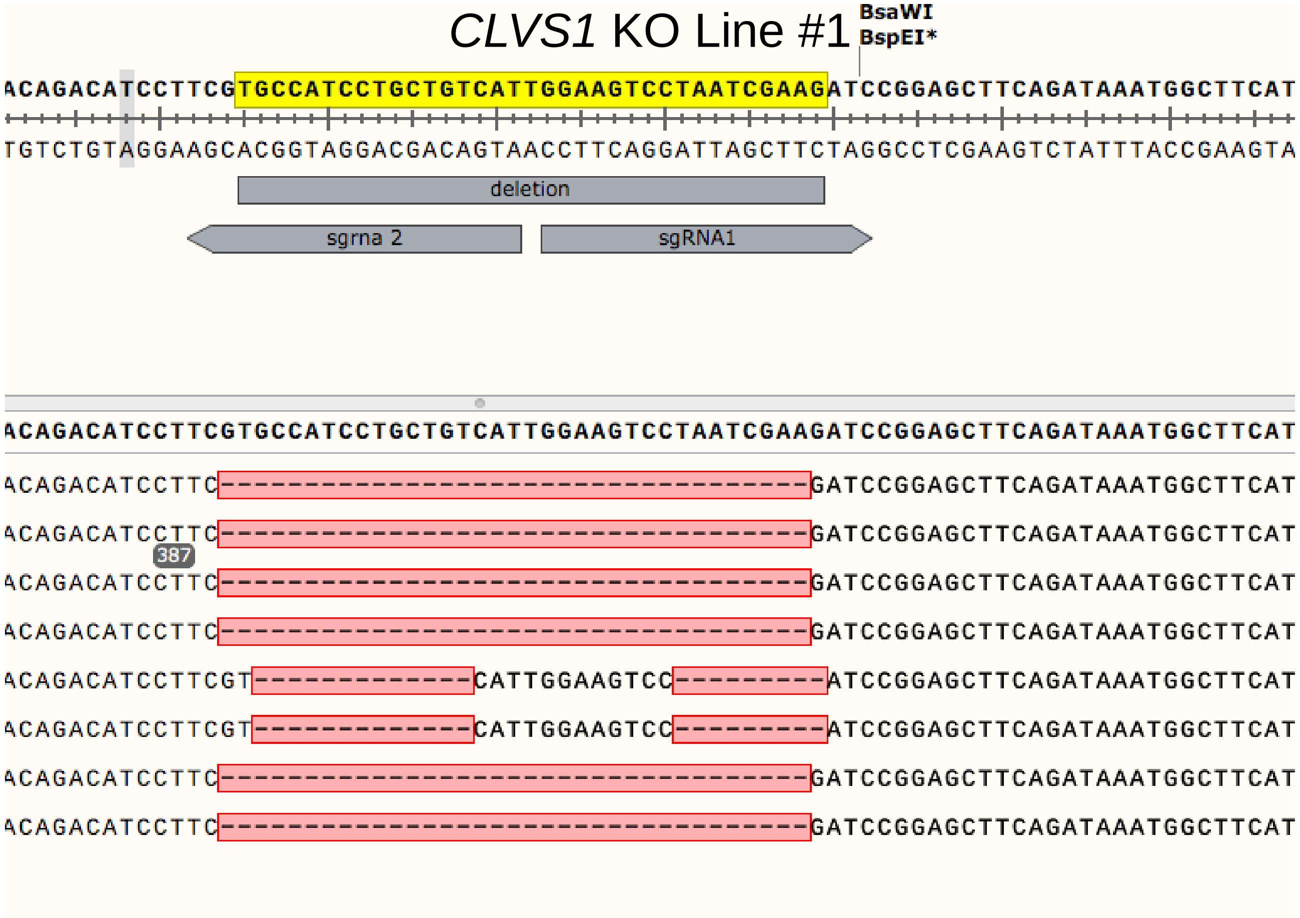

6/8 clones have an out of frame 35bp deletion
2/8 clones have a 22bp deletion

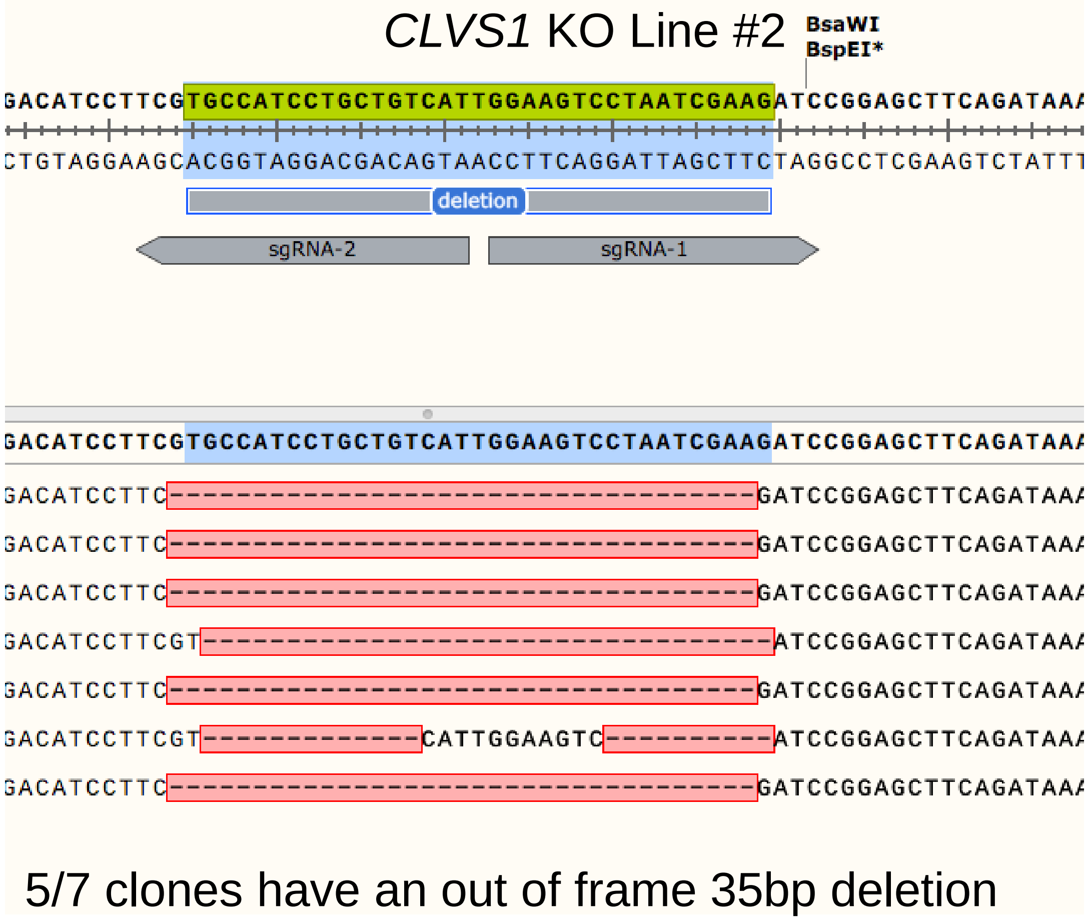

5/7 clones have an out of frame 35bp deletion
1/7 clones have a 23bp deletion
1/7 clones have a 34bp deletion

Supplementary Figure 9: *CLVS* H310Y construct

A.

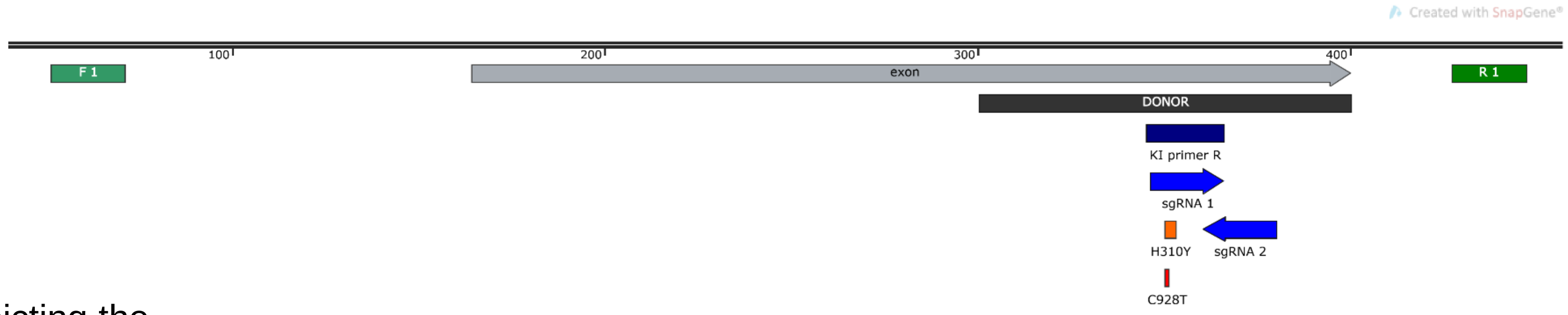

***CLVS1* H310Y KI Podocyte Lines**. **A**) Schematic depicting the targeting strategy for KI line creation with donor oligo, primers, sgRNAs to create the C928T mutation resulting in the p.H310Y variant. **B**) Sequencing analysis identified a clone with a heterozygous C928T mutation and another clone with a homozygous mutation resulting in H310Y (codon highlighted in orange). Additional sequencing identified a heterozygous *CLVS1* variant upstream of the H310Y variant in the homozygous KI line that is predicted to be benign by Polyphen *in silico* analysis (0.014).

B.

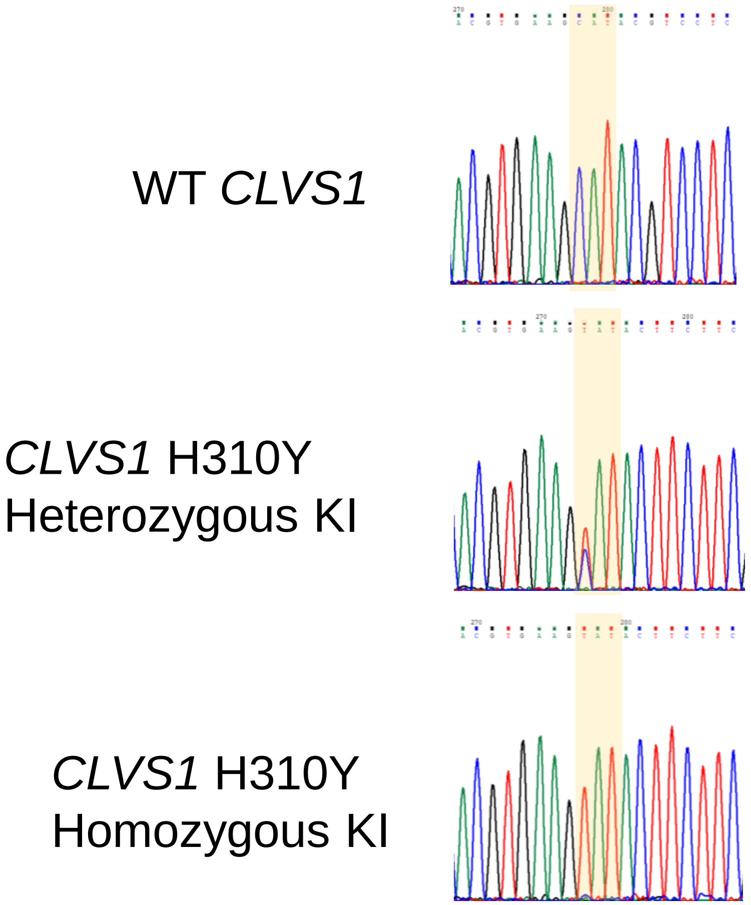

Supplementary Figure 10: Uncropped Western Blots

Fig 2

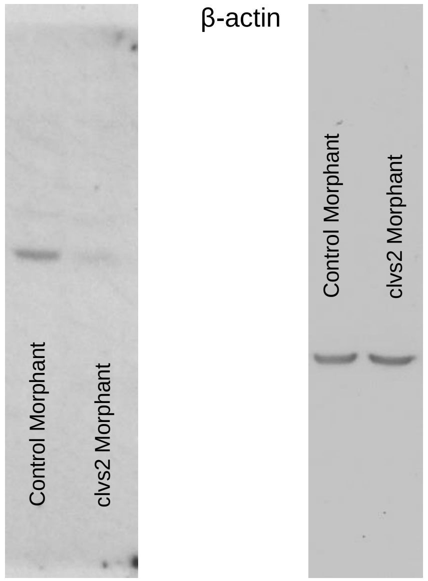

Fig 3

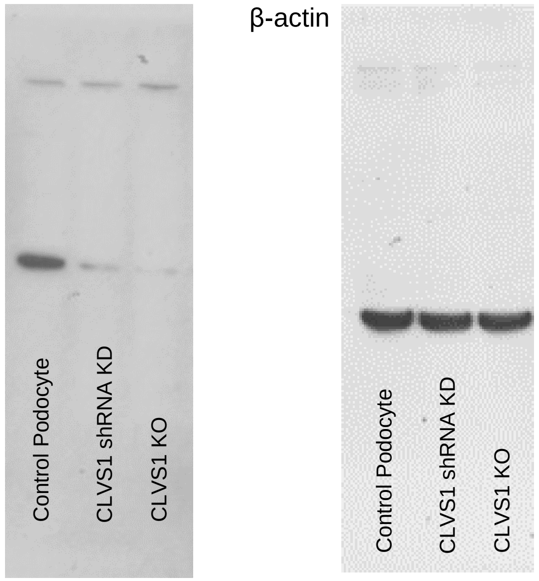

Fig 6

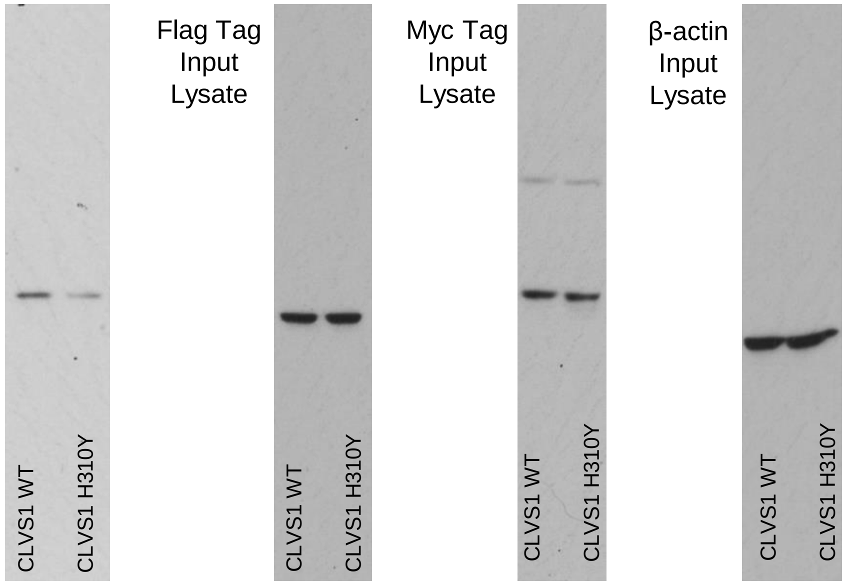

Supplementary Videos Legend

Videos depict human *CLVS1* podocyte cell lines during serum starvation with images taken every 2 hours for 72 hours. Apoptosis can be visualized when a caspase 3 substrate is cleaved and fluoresces green. Late apoptosis and necrosis can be viewed through red Propidium iodide fluorescence.

Supplementary Video 1: Control podocytes

Supplementary Video 2: *CLVS1* KO podocytes

Supplementary Video 3: Steroid treated *CLVS1* KO podocytes
